# Playfulness causes meaningful wellbeing: an investigation of 125 global playful memories

**DOI:** 10.3389/fpsyg.2026.1818085

**Published:** 2026-07-08

**Authors:** Leland Masek

**Affiliations:** Centre of Excellence in Game Culture Studies, Tampere University, Tampere, Finland

**Keywords:** culture, playfulness, positive emotion, social bonding, stress reduction, subjective learning, wellbeing

## Abstract

The role of playfulness for wellbeing has a critical and diverse relationship. In past theoretical works, playfulness as a broad concept has been studied as causing broad wellbeing meanings. In contrast, most contemporary empirical approaches to playful wellbeing focus on a narrower construct and findings. This is, in part, because a comprehensive bottom-up approach to playfulness and its wellbeing associations for individuals has not delimited a broad empirical landscape for future study especially for diverse cultural groups. This work contributes to such a question by analyzing 125 highly playful experiences reported in a semi-structured interview had by individuals from 42 national backgrounds for their primary consequential meaning. Data was analyzed qualitatively based in grounded theory and supported by micro-phenomenological stabilization techniques. They report wellbeing as the essential meaningful consequence of playfulness with four subtypes: positive emotion, stress reduction, social bonding, and subjective learning. In addition, there were three degrees of impact described: transient states, environments, and personality change. These findings reinforce a critical role for play as a broad concept effecting specific forms of wellbeing in individuals’ lives across culture.

## 1 Introduction

How do having highly playful experiences relate to wellbeing? This question has classical theories from positive psychology (Maslow, 1971a), occupational therapy (OT; Bundy et al., 2001), and psychotherapy (Winnicott, 1971). The answers these foundational works propose are also quite diverse yet essential. Playfulness is generally accepted as the “primary occupation” of children in OT (Bundy et al., 2001). Maslow wrote that playfulness was one of the “being values” that self-actualizing people have in their experience of the world (Maslow, 1971a, p. 135). Winnicott wrote “on the basis of playing is built the whole of man’s experiential existence” (Winnicott, 1971, p. 64). Contemporary theorists have also made major theoretical claims about playfulness, play, and wellbeing, such as that it reduces population-wide psychopathology (Gray, 2011) or “playful adults live an average of ten years longer than their less playful peers” (Gordon, 2014, p. 249) or that lack of play in childhood is a causal factor for mass-murderers (Brown and Vaughan, 2009, p. 25). The consistency and size of such major claims warrant empirical investigation.

Empirical studies of playfulness, have generally focused on narrower conceptions and present nuanced findings, though generally supporting a positive relationship to wellbeing. A major barrier in synthesizing these results is that “playfulness” can refer to a wide variety of concepts, with potentially different wellbeing meanings. Playfulness has been defined in a variety of ways historically, from a state of mind, a personality trait, a behavior, a mood, a design principle, or a value (Masek and Stenros, 2021). Numerous theoretical disagreements are hidden inside of different constructs including questions about whether play is “for children” (Tonkin and Whitaker, 2019) or includes humor (Proyer, 2018), creativity (Skaggs and Twede, 2023), danger (Schechner, 2017), negative emotions like suffering (Arrasvuori et al., 2011), or even torturous coercion (Trammell, 2020).

What complicates this question even more is that most authors agree that in addition to whatever the essential meaning of play is, individuals are often taught many culturally loaded norms about what playfulness should be (Besio and Amelina, 2017) such as is playing in certain ways as an adult immature? In this way, what individuals’ associate as “play” may be a culturally affected rendition (e.g., Barnett, 2017) of what they have been taught about play/playfulness rather than what they have directly experienced. This is aligned with a primary concern of micro-phenomenology called “preconceptions and theorizations about experience” (Petitmengin, 2006, p. 2). In this lens, people have two manners of sharing information about themselves: first is theoretical knowledge which is “judgments, assessments, or comments” (Petitmengin, 2006, p. 235) about how a process should have worked often according to culturally loaded education. The second manner of subjective description is the more challenging direct embodied remembering of an actual specific experience, what is called an “embodied utterance” (Petitmengin, 2006, p. 257). This places past reports that investigate playfulness purely conceptually as possibly over-investigating taught norms about appropriate play (i.e., creative or childlike) rather than actual memories of playfulness from individuals’ lives. This thus presents a serious gap for contemporary playfulness scholarship for scoping the diversity of wellbeing consequences that may come from playful experiences across culture.

This text contributes to this question by presenting empirical, bottom-up, research data on how 82 young adults, from 42 countries around the world, described a “highly playful experience” as meaningfully causing wellbeing in their life. The current text analyzes participants’ responses to the question “What is the meaning this experience has had in your life?.” Participants answers were generally discussions of *meaningful consequences* that are also essential elements of wellbeing in positive psychology. In this way, this work aims to open certain conversations on playfulness and wellbeing, reinforce certain theoretical approaches, and challenge some others. The current investigation stands uniquely as a cross-cultural comparison, with a reinforcing connection to micro-phenomenological interviews, peak experience research, and grounded theory (Glaser and Strauss, 1998). Thus, this text aims to contribute inductive insight toward the following question: How is highly playful experience, in its full diversity, self-associated to wellbeing in individual’s lives across diverse cultures?

Playfulness and wellbeing are both diverse terms with essential connections in theory and empirical confirmatory studies. There is a growing gap between two approaches in the discourse: Empirical studies on playfulness and wellbeing have generally been on narrowly defined forms of play(fulness) with diverse nuanced findings. In contrast, theoretical approaches generally understand play as a large, not comprehensively defined, concept and have been used to generate theories of *wellbeing*, especially in positive psychology. This section will overview this difference, why it’s important, and how playfulness as *engagement-priority* (e.g., Masek, 2025) enables a basis for empirical integration on playfulness writ-large.

Recent literature reviews have shown diverse empirical perspectives for playfulness as a mediator, moderator, and outcome variable in adult mental health interventions (Shen and Masek, 2024). Playfulness has been connected to a variety of effects on diverse forms of “wellbeing” across all ages (Masek, 2023b). A variety of medical disabilities are associated with reductions in childhood play such as physical disability (Besio and Amelina, 2017) or pre-natal alcohol exposure (Pearton et al., 2014). Playfulness is associated with an increased capacity to cope with stress in adolescents (Staempfli, 2007), young adults (Magnuson and Barnett, 2013) and older adults (Chang et al., 2016). In adults, playfulness has been correlated with lower rates of depression (Proyer et al., 2021), increased relationship satisfaction (Proyer et al., 2019; Brauer et al., 2023), and physical wellbeing (Proyer et al., 2018), with also moderate associations with counter-indicative components of wellbeing such as impulsiveness (Proyer, 2017). Playfulness as a personality trait was shown to be predictive of overall wellbeing of adults in Australia (Farley et al., 2021) and associated with life satisfaction and other components of wellbeing for middle and older age adults (Brauer et al., 2024; Parker et al., 2023). There is also extensive theoretical work on how playfulness is connected to public health (Tonkin and Whitaker, 2019).

Empirical approaches generally investigate specific forms of playfulness. Playfulness is often defined as either a personality trait (e.g., Proyer, 2017) or a state of mind (e.g., Webster and Martocchio, 1992) and contrasted to play as a behavior (e.g., Burghardt, 2005). There are numerous other ways of conceptualizing playfulness though, including as a design feature (Arrasvuori et al., 2011), a meta-motivational state (Apter, 1991), a value (Maslow, 1971a), or a mood (Bateson and Martin, 2013). Many contemporary empirical approaches to playfulness specifically separate it from other phenomena such as humor (Proyer, 2018) or games (Deterding et al., 2011).

In contrast to approaches that delimit differing forms of play; a broad integrative approach to playfulness has generated widely used theories for wellbeing in positive psychology. For example, Oades and Mossman (2017, p. 13), in a review highlight seven major theories of wellbeing in positive psychology and three of them studied play behaviors to generate their theory. I will expand upon the history of Csíkszentmihályi’s (1975) *Flow* theory, Fredrickson (2004) *Broaden and Build* theory, Deci and Ryan (2000) *Self-Determination* theory, as explicitly investigating play behavior to derive their theory of wellbeing.

Mihalyi Csíkszentmihályi is credited as a founder of positive psychology (Seligman, 2011) who built the foundational theory of wellbeing in positive psychology: *Flow* theory (Csíkszentmihályi, 1975). *Flow* theory was originally constructed by Csíkszentmihályi’s investigations into adults becoming fully involved in an activity without an external reward which he described in an interview as

Immediately I thought about play…. I started looking at and studying adults who spent a lot of time doing things that didn't bring them any kind of external reward: chess players, mountain climbers, long distance swimmers, etc. And… It was not like each game or each activity produced a different feeling that's rewarding. They all produced the same kind of rewarding experience. (Beard, 2015, p. 355)

This quote frames *Flow* theory as coming from investigating adult play behaviors in a broad conceptual way including sports, games, play etc. *Flow* theory has also continued to be widely used in defining measures of playfulness (see Masek and Stenros, 2021). Csíkszentmihályi viewed a broad approach to understanding people “who were *involved in play* as well as real-life activities” (emphasis added Csíkszentmihályi, 1975, p. xiii) as the essential starting point for his understanding of wellbeing.

Broaden and Build (B and B) theory is similarly an empirical approach to understanding human wellbeing that studies play as a broad concept. As Fredrickson (2004) writes, they study how positive emotions

broaden an individual’s momentary thought–action repertoire: joy sparks the urge to play…by broadening an individual’s momentary thought–action repertoire—whether through play (Csíkszentmihályi, 1975, p. xiii), exploration or similar activities—positive emotions promote discovery of novel and creative actions, ideas and social bonds, which in turn build that individual’s personal resources; ranging from physical and intellectual resources, to social and psychological resources (Abstract).

B and B is thusly also a valuable approach to wellbeing that based its empirical focus on the study of play as a wide category of behavior, including humor and music (See Fredrickson, 2013).

Finally, Self-Determination Theory (SDT) is a psychological theory on how intrinsic motivation represents a critical function for human thriving. Its historical beginning was investigating the inverse motivational effects when “college students were paid for working on intrinsically interesting puzzles,” and then broadened with “more than 100 similar studies” (Deci and Ryan, 2012, p. 417) concluding that “SDT suggests that it is part of the adaptive design of the human organism to engage interesting activities, to exercise capacities, to pursue connectedness in social groups” (Deci and Ryan, 2000, p. 229). Interesting tasks, puzzles and games are similar example phenomena to what Csíkszentmihályi and Fredrickson studied. Puzzles, word games and interesting activities are also typical examples in play behavior typologies (see Deci and Ryan, 2000). This contrast of intrinsic/extrinsic motivation is also typical for a variety of theories functionally describing playfulness as non-consequential engagement, i.e., autotelic or paratelic activities (Masek and Stenros, 2021).

These theories of wellbeing and their relationship to play in a broad sense provide an important background for the current text. Each of them started with a non-theoretically delimited and broad approach to things often considered playful. Authors investigated games, music, creativity, puzzles, interest, humor and play, with conclusions that they operated with a similar wellbeing consequence in their conditions. These widely used theories frame the need for the current work. These past works, while presenting a *broad* view of playfulness, do not present a *comprehensive* view of playfulness. The difference between broad and comprehensive approaches to play is an important one. For example, Csíkszentmihályi’s flow theory concluded that the diverse behaviors he analyzed critically had “clear goals and feedback” (Csíkszentmihályi, 1975, p. 138). This directly conflicts with some scholars’ viewpoints that “games and player communities can differently facilitate and emphasize ludic, rules-and-goals-focused gaming, or paidic, pretense-and-roles-focused playing” (Deterding, 2016, p. 108). In this way, it is possible that Csíkszentmihályi’s broad approach was still critically lacking the breadth to look at non-rules-and-goals oriented players. There is similar potential limitations in the other theories listed as well, with for example Broaden and Build’s focus on positive emotion contrasting with versions of play that have non-positive emotions such as “suffering” (Arrasvuori et al., 2011, p. 4).

This is not a criticism of the above works, as none of them endeavor to construct a comprehensive typology of playfulness and its wellbeing consequences. Thusly, while they investigate many diverse presentations of play, it is still valuable to investigate how individuals would define the wellbeing consequences of their playful experiences. If foundational theories of wellbeing are generated by investigating the consequences of playful experiences, as a broad concept, then how individuals associate their own self-labeled playful experiences with wellbeing is a valuable question to comprehensively address. This speaks to the value of a bottom-up investigation, where instead of a theoretical framework being chosen first and then subsequently used to analyze data (i.e., top-down or a deductive framework), inductive and abductive analysis is used to create a more general theory based upon the patterns within the dataset (Haig, 2013).

Past scholars have conducted qualitative bottom-up investigations for functions of playfulness with supportive findings for the current work. Barabadi et al. (2022) investigated 38 English as a Foreign Language learners from Mashhad Iran on the perceived functions of playfulness in that learning context constructing “four categories: Fun and laughter, creativity, mastery orientation, and cultivating relationships.” Proyer (2014) conducted a questionnaire with 324 lay people reporting seven categories of answers for the functions of playfulness as “(1) well-being; (2) humor and laughter; (3) mastery orientation; (4) creativity; (5) relationships; (6) coping strategies; and (7) coping with situations.” (Abstract). These works both explicit unpack wellbeing relevant findings, and also fundamentally take a different approach to the topic of playfulness. These two past studies investigate the function of “playfulness” as a conceptual term in individuals’ lives, whereas the current work asks for the consequential meanings (i.e., functions) of specific memories of playfulness. This difference is discussed well by Petitmengin (2006) who separates “intellectual memory, based on conceptual knowledge, which is not linked to a specific lived experience, with affective memory, which enables the rediscovery of the past” (Petitmengin, 2006, p. 244). Thus, in this way, it is also useful to gather data not just about conceptual playfulness, but also about effects of specific experiences that represent playfulness.

This work thusly is not about what playfulness *is* in theory but rather what it *causes* in particular human experiences. In this way, it is most beneficial to integrate the work into a broad, yet defined, theory of playfulness. This work integrates with a contemporary approach to playfulness through the lens of *engagement-priority* (Masek, 2024). Playfulness as *engagement-priority* defines playfulness as all phenomena where *how* it operates seems to prioritize action, emotional reinforcement, and/or social connection (i.e., *engagement*) while not having any *serious* priority that is more definitive (i.e., *reality, conventions, or consequences)*. For example, a board game is often playful in this lens as it is an *activity*, with *emotional reward*, that is usually *socially shared and* is primarily done for those elements. This model was originally introduced to make sense of interdisciplinary scholarship on playfulness and concluded it was an organizing principle where “Playfulness prioritizes engagement over external consequence, realness, or convention.” (Masek and Stenros, 2021, p.23). It has been further reinforced in interview studies where participants from a diversity of cultures similarly typified their personal view of playful experiences as engaging (active, emotionally reinforced, and socially shared) with that being the priority (not too serious) and with the general description of “An active experience that you like, share with others and is not too serious either in manner or context” (Masek, 2024, p. 871).

This framework is thusly useful as a context for the current work as it provides a theoretical connection for a diverse set of phenomena associated with play, having been applied to playfulness across culture (Masek in press), video game player motivations (Vahlo et al., 2022), rule-free play vs. rule-bounded play (Masek, 2023a), humor (Masek, 2025), and social parties (Masek and Stenros, 2024). This theoretical approach is also critical context as the interview dataset from these past works is also the basis for the current bottom-up investigation. Taken together this work contributes data to the research gap of how the consequences of highly playful experiences may be connected to wellbeing as reported by a diverse cultural group of people.

## 2 Materials and methods

This study used two primary frameworks to create a triangulation of methodologies (Thurmond, 2001) and guide the creation of a semi-structured interview protocol (Kallio et al., 2016) on highly playful experiences and how their meanings are associated to wellbeing by respondents. The first framework was *Grounded Theory* (Corbin, 1997) and the second was *Micro-Phenomenological Analysis* (Petitmengin, 2006). Both methodologies were influential and adapted during this study. Data was processed using a five-phase thematic analytic technique proposed by Yin (2015). This analysis is a descriptive phenomenology project, which intends to present participants’ own views of their experiences.

This work aims to build methodologically on previous works that study playfulness qualitatively. Whereas past studies have looked at qualitative views of “playfulness” as a general concept (e.g., Guitard et al., 2005; Lubbers et al., 2023; Masek, 2025), this work specifically is more interested in investigating patterns that occurred in individuals’ lives, from specific playful memories and meanings. This choice is in part chosen based on Maslow (1971a) Peak Experience research. Peak experiences have been used to gather data on various complicated concepts across culture before including how individuals experience love and passion (Mouton and Montijo, 2017), wilderness (McDonald et al., 2009) and happiness and joy (Scott and Evans, 2010). A pilot interview was used where the first 10 interviewees were asked about a “peak playful experience”. Interviewees often did not know what a peak experience was, thus subsequent interviews remained predominantly the same but instead individuals were asked to select their memory based on the more commonly understood language of “highly playful experience”.

Grounded Theory (Strauss and Corbin, 1997) guided the choices for bottom-up elicitation of participant’s interpretations and ongoing theoretical development categorizing those views across a theoretically chosen participant pool. Grounded theory was selected as it is one of the foundational theories guiding qualitative research in social sciences (Glaser and Strauss, 1998). It is valuable as a set of tools for seeking to understand phenomena from a participants’ perspective. Specifically, the following elements of grounded theory methods were utilized: constant comparative analysis, open and intermediate coding, theoretical sampling and saturation, theoretical integration of codes and categories, and concurrent and continuous data generation and analysis, and theoretical sampling (See Teppo, 2014).

Micro-phenomenology was the second guiding framework, used primarily for enabling interviewees to recall complicated elements of specific memories and more accurately deconstruct important sequential aspects of those memories. In specific, the following interview stabilization considerations were applied: stabilization of attention, focusing upon a singular experience, refocusing from what to how, focusing on different dimensions of experience, retrospective analysis (re-enactment), and scale of precision (Petitmengin, 2006).

To integrate these two guiding methodologies, a five-phase qualitative thematic analysis technique was used to connect and interpret individuals’ specific utterances across interviews (Yin, 2015). This work presents the results to the question “What is the meaning this experience has had in your life?.” Answers to this question firstly compiled into a larger database (step 1). The answers were disassembled for representative quotations for different ideas expressed with summary phrases attached for each quotation (step 2). These quotations and fragments were reassembled into larger explanatory categories (step 3). Fourthly these categories were interpreted for what they seemed to be expressing in essence and finally (5) This report was written to explain the paradigm.

### 2.1 Procedures

The data set was gathered at a Finnish University from spring 2019 through winter 2021 where international students from a diversity of backgrounds were asked to share a memory of a “highly playful experience” (HPE) which was defined as

please think of playfulness as the internal experience often associated with play. Now, imagine playful experiences and try to think of one of your most playful experiences. Because this is an internal experience you could be doing any type of activity as long as you were feeling or thinking in a highly playful way. (Interview Protocol).

The interview was broken into five sections. First, Interviewees were asked to “Please describe the playful experience in detail, focusing on the way in which you perceived and interpreted the event.” In this first section, few questions were asked by the interviewer, but rather only the interviewees’ own interpretations of the experience were elicited. In the second section of the interview, the interviewee was asked to go back to the beginning of the experience and answer specific questions about the experience focusing upon objective details in the experience such as location, timing, activities and people present. Thirdly, participants were asked about important theoretical concepts from academic literature on playfulness, including motivation, meaningfulness, rules, competence, relatedness, autonomy, perception of reality, locus of control, cultural meanings, and consequences associated from their experience to their larger life. In the fourth section of the interview, interviewees were asked to rate themselves for how playful they are, using the SMAP *Short Measure of Adult Playfulness* (Proyer, 2012). A fifth section of the interview furthermore asked participants to rate their experience using a series of scales representing the author’s developing model of playfulness, to enable the further comparative refinement of the conclusions, a standard in grounded theory (Strauss and Corbin, 1997). At the end of each interview, participants were asked if they had a second experience that they would term highly playful but also somehow contrasting to their first experience. If they did and agreed, they were then interviewed a second time focusing on their second experience, resulting in 125 total experiences from 82 interviewees.

The analysis for this work focuses on the word “meaning” specifically the question “What is the meaning this experience has had in your life?” (see Supplementary materials). Which if interviewees had any questions about this, the question was reframed as “What kinds of larger take aways or consequences did this experience have for you in your life?.” In addition, any time participants used the words “meaning” or referred to a meaning from their experience in other parts of their interview, this was considered relevant for inclusion into the quote database.

### 2.2 Participants

Interviewees were selected with the goal of having a diverse national representation as a theoretical sampling method (Teppo, 2014), as well as recommendations from past interviewees. The final data analyzes 125 playful experiences, had by 82 interviewees from 42 countries, across six continents around the world, 56 occurring in-person, 26 online, taking 22–257 min (Avg. 64 min) per interview. These experiences were primarily from young adults with the average age of interviewee being 26 with a range of 18–39. There was a moderately diverse self-described gender background with 34 women, 45 men, 1 non-binary, and two participants choosing to not disclose their gender. The national background of the participants was diverse, with representation from every inhabited continent including Asia (*N* = 31), Europe (*N* = 30), South and Central America (*N* = 7), the US and Canada (*N* = 7), and Africa (*N* = 7). For a full list of national backgrounds, see Supplementary materials.

The interviewer was also an international student at Tampere University and recruited individuals living in the same international housing complex, or through a public, international coworking and game space on campus. All interviewees were asked in person for their participation. They were provided a brief in person description of the nature of the study, sent a data protection document and consent to participate form linked in Supplementary materials with a more extensive description of the studies purpose. The study followed data protection and ethical oversight processes in alignment with the laws of the country of implementation. This recruitment was not randomized but rather a purposeful sampling (see Suri, 2011), seeking participants from diverse national backgrounds. This study’s participant recruitment is also aligned with ethnographic approaches of a researcher asking available participants who have access to specific cultural knowledge (Fielding, 2008).

### 2.3 Data analysis

All interviews were transcribed verbatim and read before being analyzed. The data analysis is ongoing and currently includes almost 2,000 distinct codes interpreting the data and numerous axial codes and resulting categories. The data has been fully anonymized, and quotations will be reported with an experience number, with no gender or age information, unless the participant themselves thought this was critical to understand the experience.

The resulting categories are based upon the theoretical generation process recommended by grounded theory (Strauss and Corbin, 1997). Grounded Theory is a bottom-up research method that prioritizes individuals’ own perceptions, and “begins with inductive data, invokes iterative strategies of going back and forth between data and analysis, uses comparative methods, and keeps you interacting and involved with your data and emerging analysis.” (Charmaz, 2014, p. 1). Interviews were conducted in an iterative fashion of approximately 10 interviews at a time followed by data analysis. The interviews were not analyzed for each participant’s intended meaning, with codes and categories meant to describe what an individual was describing as important in a line, or in a series of sentences.

Coding first occurred in a line by line fashion, whereby each time a new critical idea was introduced by the interviewee, a new code was created, then focused coding of larger pieces of data, then axial coding of different categories that shared similar features, condition, actions/interactions, and consequences, then finally theoretical coding of how these experiences fit into a larger theoretical framework. Codes were in a *constant comparative analysis* (as recommended by grounded theory) between different interview transcripts, levels, categories, and theory. Theory was developed each time an iterative cycle of interviewing occurred. Memos were also written throughout the process.

Micro-phenomenology (M-P) was used as a guideline for the constructing of the interview protocol to enable interviewees to have deeper and clearer recall of their highly playful experience. There are five methodological goals that M-P most essentially tries to solve that influenced this study:

to stabilize his attention on the experience described,

to convert his attention from the ‘what’ that usually absorbs his interest to the ‘how’,

to move from representations and general beliefs about the experience in question to the description of a singular experience,

to direct his attention toward the different dimensions of his experience,

to be more precise in his description” (Petitmengin, 2006, p. 255).

M-P also has specific criteria to measure success of an interview dataset (Petitmengin, 2006, p. 257–258). To verify how many participants were able to engage in the admittedly high standard that M-P holds, the four verification measures described above were used. By analyzing the transcripts, 75 out of 125 experiences (60%) were detailed in a fashion to satisfy all the described criteria of M-P and thus be considered “an embodied utterance” (Petitmengin, 2006, p. 257), where participants were likely to have been reliving the memory sufficiently as an embodied state rather than telling a story about the memory from a more removed mental position. In this way, micro-phenomenology was used as a set of stabilizing tools, not as a fully satisfied method.

By analyzing these 125 playful experiences, this work intends to describe how playfulness occurred in everyday life by people from diverse cultural backgrounds and how they viewed these as connected to their wellbeing. As much as possible I will aim to use the words of the interviewed subjects. Analysis is focused upon words and meanings that multiple interview subjects were discussing.

## 3 Results

By analyzing 125 HPEs participants described the “meaning” of playfulness generally as having wellbeing consequences in their life. Four themes will be presented in the work: Positive emotion, Stress Reduction, Social bonding, and Learning. In addition, participants described different degrees of impact playfulness had in relation to wellbeing: a passing experience of wellbeing (state), an enduring repeated context for wellbeing (environment), or a fundamental change in their approach to life (personality).

This language of degrees of impact is taken from past scholars of playfulness. States of mind in this work refers to “affective or cognitive episodes that are experienced in the short run and fluctuate over time.” (Webster and Martocchio, 1992, p. 203). Thusly, this will be used to describe participant reflections of a short-term affective or cognitive consequence from their playful experience with little described repetition. Environments of playfulness derive from an interactionist framework description that “environment is the general and enduring background” (see Shen, 2020, p. 540) of play. In these environmental descriptions, participants discuss the consequences of playfulness as stable results of external factors such as activities, people, places, times etc. Finally, personality consequences of HPE’s are defined through playfulness as an individual differences variable frame (e.g., Proyer, 2017) or when the impact was described as an “orientation that transcends situations and activities” (Shen et al., 2014, p. 59).

To make the report as clear and readable as possible, the results will be reported via the four categories of wellbeing meanings, and then how each wellbeing meaning presented itself as a state, environmental perception, or personality trait and will be unpacked within that section. Finally, these reported wellbeing meanings will be analyzed for how they are integrated with the seven major theories of wellbeing in positive psychology as identified by Oades and Mossman (2017, p. 13) as well as other classical theories of wellbeing.

The current findings will focus upon overlapping frameworks of playful experiences across culture rather than contrasting them. How culture is intertwined with playful experiences is a topic for a different article (Masek forthcoming). Of note, there are no clearly emergent patterns for how participants from differing cultures had different wellbeing associations. Bottom-up qualitative methodology is also not typically used to contrast frequencies of different groups, so this work is more integrative and descriptive. Each of these sections will be written firstly with a description of the bottom-up data the defines then and then integrated with reference to past scholarship that would describe a similar role for playfulness and wellbeing. Due to full anonymization, quotations are reported based on experience number and identity factors of the respondent will only be described as broad categories if the person themselves viewed it as essential.

### 3.1 Positive emotion

One common interpreted meaningful consequence of a highly playful experience in participants’ lives was experiencing a positive emotion. The most common types of positive experiences were joy, happiness, fun, or being seen as one of their best memories in general. Positive experience was in general described as an opposite to stress, suffering, or any type of unwanted experience in life.

Highly playful experiences were described as creating a short-term positive emotion. There was a variety of positive emotions described such as and experience spraying friends with water as “the game itself was just extremely fun” (E2), or a memory of playing basketball as “it’s just a time that I was really enjoying.” (E98), playing cards as a child having the meaning “just happiness” (E54), or watching a humorously bad movie with a family member as “it was a good experience,” (E41). In this way, we can see an emergent pattern of highly playful experiences being reported as causing a short term mental state of positive emotions.

Other participants described this positive emotion as a fixed element of a repeated environmental interaction, for example describing their weekly board game group as, “it is important, because it’s like the time we spend with my friends playing, I really enjoy. And it’s like, it’s the little moment of the week… like, two hours that we are playing and having fun.” (E25). Others reported that specific games, such as a social party/drinking game was consistently causing a positive experiences saying “Yeah, just like, it’s, it’s one of my favorite games. And it’s just an opportunity to be playful, and to have fun. And that goes back to, like, my ideal day is just spending time with people that I love, and just having, like a fun game with them. And this is a way that I can do that.” (E32). In this way, participants described highly playful experiences as specific environments, whether a specific game, social group, or time, that created positive emotional experiences in their life.

Other participants described their HPE as influencing their general approach to life so as to create more positive experiences in general. Participants described the meaning of HPE’s such as an annual tradition of playing Volleyball with family members as “It influences the experiences that I seek out now in everyday life. So like in any situation, I want to just have fun” (E31). Other respondents framed a similar broad identity effect such as playing an impromptu water gun fight with children as “it like really made me like get back into that, like childish mindset of like, life is literally you can do whatever the f you want.” (E9).

Positive emotional consequences for playfulness have broad representation in theories of wellbeing. Maslow listed playfulness as a perceived being-value with similar example words writing “Playfulness: fun; joy; amusement; gaeity; humor;” (Maslow, 1971a, p. 135). Apter (2001) definition of paratelic or “arousal that they [activities] produce can be so enjoyable that we seek out and construct them” (p. 23) would be quite aligned. Broaden and Build theory studies positive emotion in its essence (Fredrickson, 2013). It seems similar to how Flow theory describes the justifying “rewarding experience” of certain activities (Csíkszentmihályi, 1975). Positive emotion is the first criteria of the PERMA model of wellbeing (Seligman, 2011). Positive emotion as described by participants is highly similar to how SDT conceptualizes “interesting” activities (Deci and Ryan, 2012). In this way, we see a major confirmation that highly playful experiences are seen, from individual’s own memories as major contributors to positive emotional experiences.

Wellbeing effect 1: *Highly playful experiences* are reported as causing *positive emotion*.

### 3.2 Stress reduction

Highly playful experiences in participants’ lives were secondarily associated with a reduced perception of stress or negative emotions. These emotions were most often fear or feeling controlled. Several reduction and relief from negative emotion was at times described as being replaced by a playful positive emotion as described in the previous theme.

States of mind that relieved stress included individual moments of high stress that were relieved by a highly playful experience. For example, an interviewee discussed going to a roller coaster and its meaning for their life being “it’s really important experience for me because I remember before we go into the amusement park, I was really stressful with all the schoolwork in my internship projects, but after that after doing the kinds of activities I feel like my stress is released. And I can face my reality more with more energy.” (E81). There were several experiences that reduced the sense of stress a person felt including climbing a mountain relieving the stress caused by cultural expectations of academic success (E95), going out socializing with friends reducing the stress of a breakup (E80), or lowering fear about being publicly known as dating homosexually (E49).

For other participants, this reduction in negative stress was a known repeatable aspect to the activity or environment of play. This was most often associated with physical play acts such as one participant describing the meaning of childhood sports as “My body will be working but mind will sort of rest and refresh” (E15). Other participants similarly described going on a jog while imagining being chased by zombies as having the meaning of “On the days that I’m super stressed… Like when you do a restart with a computer. I just let my mind flow on the run, I finish [the] activity and I start to think again, it’s like, okay, now it seems better,” (E26). Similarly, participants described highly playful experiences causing a relief in “stress,” such as spending time with friends lowering stress from university work (E25), playing videos games reducing feelings of loneliness (E45) and table-top role-playing games reducing the experiences of depression (E56).

In addition, highly playful experiences were sometimes reported as symptomatic of, or causing a change in a generally stressful lifestyle, usually discussed as “not taking things too seriously.” For example, one participant discussed lifelong joking in their family, and specifically jokes made with a family member who was hospitalized as having the meaning of “I think that it’s that you can never take anything too seriously. Like, you can always find the positive.” (E123). Others similarly described not taking things too seriously as a part of their personality, as for example a teacher willing to jokingly mock students (E113), or someone MCing a wedding being evidence of how “Meaning, well, I sometimes believe we should not take life too seriously.” (E77) or that going to a flirty networking event made them in general less awkward and instead more “comfortable with being human” (E21). In this way, we see individual’s describing a generally lowered level of stress, or at least a reduction in how defined by that stress they perceived themselves to be.

Stress reduction was also reportedly tied to risk-behaviors. As one participant described participating in a highly playful final dance performances’ meaning as

if I consider my choices before the concert, like, abandoning this health situation, I would really say whether I need to do that or not, if I [would] do it now, I mean, like, because it was kinda like, serious… the problem” (E53).

In this experience, the playful experience was reported as lowering the perceived stress or seriousness of a health situation, which the participant later regretted. In this way, some participants described playful experiences as defined as taking life *too unseriously,* for example, one participant who similarly regretted a playful experience in a workplace, where they pretended to be doing work, as “It was not taking the priorities seriously. So, I think that’s what describes playfulness” (E79). Most risk behaviors were seen as worthwhile by participants though. Participants said playful experiences included many risks they would not do otherwise such as risking law-breaking (E2;4;13;17) or their health (E14;15;61), typified by reports such as “I was doing like crazy things, and I wasn’t caring about consequences.” (E14), or socially risky behaviors where participants’ playful sexting experience having the risk that “[authority] is going to kill her if she ever finds out that she’s has gone against [religion] morals and started an immoral [sexual] relationship” (E46). Put together some individuals were sometimes inhibited from taking certain risks because of stress and playfulness lowered the perception of that stress, thus increasing their risky behaviors. In general, this increased risk tolerance was seen as a positive, but not always. This data aligns with findings that playfulness is associated with impulsiveness (Proyer, 2017).

The relief of stress is aligned with past theories of playfulness and wellbeing. Maslow’s conception describes playful being-values as “effortlessness” (Maslow, 1971a, p. 135) which would align with how participants described reduced stress. This category would also align with the host of studies on playfulness improving stress resilience (Chang et al., 2016; Magnuson and Barnett, 2013; Staempfli, 2007). In this way, playfulness was reported as meaningfully lowering stress, which in general is seen as positive while also increasing certain forms of risk.

Wellbeing effect 2: Highly Playful Experiences are reported as being meaningfully reducing of stress and negative inhibitory emotions and thusly also increasing certain types of risk.

### 3.3 Social bonding

Another commonly reported meaning for highly playful experiences was social bonding with the people involved in the playful experience. This was once again present as a temporary experience of bonding, a sense of community caused by a playful environment that persisted with specific people, and as a long-form skill or preference for bonding in general. This meaning was closely aligned with the term “friendship” with several respondents discussing their most significant friendships being, in their essence, playful and associated with highly playful experiences.

As a state of mind, playfulness was described as inducing a short-term sense of social bonding with those people in the experience. As one participant described a highly playful experience dancing at a concert as creating a “sort of hive mind… connecting with people. Maybe just like briefly” (E3). Others discussed a meaning of social bonding as a temporary effect of university welcoming rituals (E1), playing in an elite ping pong tournament causing bonding with the opposing player (E10) or escape rooms causing family-bonding with those playing (E93).

The social bonding meaning was also sometimes described as a repeated element of an environment, often a specific game played many times. One participant described playing outdoor games as a teenager as meaningful because “the game makes your friends closer to you” (E15). Frequently respondents would describe permanent friendships because of their highly playful experiences for example wrestling during a summer vacation as “I’d say the bonding is the most important part. Yeah. Like I feel more connected to these friends who I wrestled with,” (E38). Others described permanent friendship from so-called playful “shenanigans” of recreating a joust in a dorm hallway as having “really built our friendship up to what it’s still today” (E17). Participants had playful experiences repeated as bonding rituals both weekly with board games (E25) and annually such as holiday bonding rituals playing board games with family members (E18) or open world video games with friends (E83).

Finally, participants would describe highly playful experiences as increasing their general interest or skills in social bonding. For example, one participant described playing sports with their school-friend group as changing how social they were in general saying “That’s the turning point, I tried to talk to people more. In my, like my elementary school, I was so introverted, but still, I could not talk after the football match and something. But in my high school, I have those friends and those friends have so many friends, other friends and classmates” (E60). Another participant discussed how an annual volleyball game with family has affected them because it “influences the experiences that I seek out now in everyday life… I’d rather just find my people, my community, and just have fun with them.” (E31). Playful experiences such as inside jokes (E125) and board gaming (E43) were described as important for maintaining long term romantic relationships. Other respondents similarly described the meaning of the HPE as causing or representing their skill of socializing during playful political discussions (E110) or helping them get the ability to to talk to people socially in flirting (E21), student parties (E50) or university welcoming rituals (E71).

Social bonding is a widely studied critical component for human wellbeing. Seligman’s PERMA model includes “Positive Relationships” as one of the five primary criteria of wellbeing (Seligman, 2011). Broaden and Build typifies play as developing uniquely social bonds and social resources (Fredrickson, 2013). Self-determination theory defines “relatedness” as one of the three psychological needs that explains intrinsic motivation of internalization and integration “need for relatedness –that is, the need to be close to, trusting of, caring for, and cared for by others” (p. 421). In this way, the current study aligns participants self-reports on how playfulness is meaningful to past theories of critical elements of wellbeing in positive psychology as associated with play through social bonding.

Wellbeing effect 3: Highly Playful Experiences are reported as being meaningfully socially bonding for those in the experience together.

### 3.4 Subjective learning

The final widely reported meaning behind playful experiences was subjective learning. Subjective learning was a reported consequential increase how a participant felt they understood how to be in the world. In transient states it was generally a symbolic moment of creativity or learning about “possibilities” of a situation. Environmentally, participants reported learning a playful skillset or a preference for a particular type of play. Finally, sometimes participants describe themselves as learning a larger value of theirs in life, that applies across situation, such as the importance of hard work.

Participants described subjective learning as sometimes a specific non-repeated element of a memory. For example, one participant described one of their first work-place events as playfully having the meaning they “learn new things” (E5). Others described their HPE as symbolic of a learning-moment or discovery such as an unusually humorous player-made level in a game as “an exhibition of the creative possibilities of gameplay” (E28). Others described living abroad for the first time as meaningful because “I feel like it has this feeling of me figuring out this new thing like that I think I should have known but I do not.” (E91).

Respondents would describe these learning meanings as essentially learning about a playful environment in a deeper fashion. One participant described a final university project as “I learned a lot about myself. And I would say that I mean, I never thought the possibility of writing code and doing some music, which was quite surprised for me.” (E68). Another participant discussed how building with toys in childhood similarly was “Educational from the mechanical point of view, but also from a social point of view.” (E117). Participants similarly described their HPEs as teaching them about other specific environments such as playing outdoors teaching them to value nature (E6), final projects teaching writing skills (E75), sexting teaching a new preference for sexual role play (E46). Other self-learning taught how they can realistically play in a certain environment. As one woman described her HPE’s meaning as

the main meaning is that you should find, if you are a [country] or like a South Asian girl, you should find a husband who lets you be yourself, because we don't have any other way to get out of it, to be honest. I did try, just after my exams, I tried to go to [Western country] to do my bachelors and my parents were against it, like, you're a girl, alone, I can't let you go alone, I'll get married to somebody and go. So, this experience I should have had like a single person, I had to have that as a married person with a child, because my parents weren't allowing it. (E72)

In this way environmental subjective learning was at times learning how oneself can have playful experiences in a specific way.

Finally, participants described HPE’s as teaching them larger elements of life that apply to many different contexts. For example, one participant described an online boardgame experience with a particularly right-wing and manipulative opponent as meaningfully “reducing my belief in meritocracy” (E96). These types of learning were often associated with a variety of values in life such as camping with friends teaching the value of doing things in proportion (E106) or traveling teaching life values in general (E61), or theater teaching how to be confident (E47). Several participants described the HPE as meaningful as it taught them about the rewards of persistent, dedicated effort toward a goal. Such as a playful final work project meaning “It showed me that I’m a very hard-working person and that I’m also committed.” (E86) or playfully asking someone out for a date as “if you are really wanting to do something, that if you would perform, put the effort to that and you can do that.” (E92) or weightlifting teaching “discipline” (E107) or school sports teaching “work ethic” (E73).

Others described learning skills toward other wellbeing effects of playfulness. This usually presented as a self-learning how to enable wellbeing in life, as a skill. Participants described as learning how to socially bond with others during flirting (E21) in MCing (50) University welcoming rituals (71) and political discussions (110). Others described humorously playing music teaching them how to have a positive experience in general (E22). Wrestling with friends was described as teaching how to engage in a less stressed way as “it taught me also to be a sort of a little bit more spontaneous in this sense.” (E38).

Learning about one’s preferences in the world has some strong connections to past theories of wellbeing and play. This particular form of learning is described well by Maslow who writes

Thus if one thinks in terms of developing the kinds of wisdom, understanding, and life skills that he would want, he must think of what I call intrinsic education, intrinsic learning; that is, first, learning to be a human being in general, and second, learning to be this particular human being (Maslow, 1971a, p. 150).

This form of learning is also like Winnicott’s transitional phenomena as “the exciting interweave of subjectivity and objective observation” (Winnicott, 1971, p. 64). This category also aligns with Broaden and Build theory’s claim that positive emotions “promote discovery of novel and creative actions, ideas” (Fredrickson, 2004, p. 1367). This form of learning aligns with what SDT studies as increases in perceived *competence* in an environment (Deci and Ryan, 2012). These experiences would also align well with *Flow Theories* description that people should have a perceived level of skill to match the challenges of the situation (Csikszentmihalyi, 1975). In this way, we can see a form of wellbeing reported as a meaningful consequence of highly playful experiences:

Wellbeing effect 4: Highly Playful Experiences are reported as causing subjective learning about the self’s relationship to the world.

## 4 Discussion

By analyzing 125 playful experiences that have occurred by individuals from 42 national backgrounds, a picture of playfulness and wellbeing emerges. In general, participants viewed playfulness as having four forms of wellbeing-relevant consequential meanings: high positive emotion, relief from stress, social bonding, and self-learning. These four categories were framed in three major ways: as brief, transient experiences or states, as an experiential relationship to a specific environment, or a change to their sense of self across multiple contexts. This text also aligned these four wellbeing effects as essential to numerous theories of playfulness and wellbeing in positive psychology. This opens the door for a variety of interventions and confirmatory studies on what behaviors, in what contexts, and for what durations trigger these effects in individuals. Participants who had repeated events of a specific playful type were more likely to report this having a major impact on their life, but the exact duration and repetition required for varying results should be investigated. In summation, Table 1 is a visualization of key findings of this work with representative quotes.

**Table 1:** Meaningful consequences of HPE’s by category and type.

Category	State of mind	Environment	Personality trait	Relevantly associated theories
Positive experience	everybody’s just just happy and excited about it happening. And it’s very contagious. Okay, you just cannot stop smiling (E5)	It’s like, it’s the little moment of the week… like, two hours that we are playing and having fun. (E25).	It influences the experiences that I seek out now in everyday life. So like in any situation, I want to just have fun. Like that’s what matters. (E31).	Being Values of Playfulness (Maslow, 1971a), Paratelic (Apter, 1991), Positive Emotion (Fredrickson, 2013; Seligman, 2011), Autotelic (Csíkszentmihályi, 1975), Intrinsic Motivation (Deci and Ryan, 2012)
Stress reduction	I’ve been going through some really grim times and I think having that made me feel really relaxed afterwards. it’s I love playfulness. (E56)	After doing the kinds of activities I feel like my stress is released. And I can face my reality more with more energy. (E81)	Meaning, well, I sometimes believe we should not take life too seriously. (E77)	Effortlessness (Maslow, 1971b) Impulsiveness (Proyer, 2017), stress resilience (Staempfli, 2007; Magnuson and Barnett, 2013; Chang et al., 2016)
Social bonding	I remember feeling such an innate sense of wholeness, and like fitting in (E8)	I’d say the bonding is the most important part. Yeah. Like I feel more connected to these friends who I wrestled with, (E38)	That’s the turning point, I tried to talk to people more. (E60)	Positive Relationships (Seligman, 2011). Social Building (Fredrickson, 2013). Relatedness (Deci and Ryan, 2012).
Subjective learning	I feel like it has this feeling of me figuring out this new thing (E91)	Educational from the mechanical point of view, but also from a social point of view. (E117).	It showed me that I’m a very hard working person and that I’m also committed. (E86)	Intrinsic Learning Maslow, 1971b) Transitional phenomena (Winnicott, 1971), Broadening mindset (Fredrickson, 2004), Competence (Deci and Ryan, 2012)

These wellbeing meanings bear important considerations for discussion in terms of how they intersect across category. The increase in positive emotion, and stress reduction could both be seen as playfulness being an emotional regulatory (Gross, 2015) tool in certain individuals’ lives. Some participants did intentionally used playful experiences to change their or others emotional experiences. This is not definitive of all however, as some respondents had these emotional changes, but they were unintentional.

These themes also bear interesting connections to certain topics from playfulness research that are important to discuss in terms of their similarities, but also key differences. For example, playfulness literature often focuses on “intrinsic motivation” (i.e., Deci and Ryan, 2012), and this work complicates the equivocation of playfulness and intrinsic motivation. Playful experiences often feature an internal *perceived locus of causality (I-PLOC)* (Deci and Ryan, 2012, p. 420), where participants see outcomes in the environment as contingent on their behavior and expressions of their autonomy, but other experiences forcefully reject that. For example, one participant was forced to engage in a poetry reading competition and described it as

“It was not my choice to make. I could have said no though…they [the teacher and the class] were just striving me to go forward, striving me to do better. That whole experience is pretty important, and also playful in the same way…. when I didn't win as well, I lost and I felt like I let people down. But it was a part of the experience as well. And I heard good things from my like, you know, really touching things.” (E97)

This experience captures an important contrast to I-PLOC where not only was the playfulness not placed as internally caused, but the positive experience was also described as external. Social pressure was normal in experiences, and some experiences were described as not freely chosen such as reports that joking at work had the result “I felt addicted” (E79). Even more importantly, a lack of control was sometimes described as a source of enjoyment in the larger interview such as one participant who described being playfully kidnapped by their friends in a game as “I did not know what to expect, I did not know where we were going. And that made me nervous, because it wasn’t something I could control. But also, because of that, it felt really fun” (E66). In this way, playful positivity should not be assumed to always be perceived as internally caused. Future studies should investigate further the implications of externally caused playfulness.

The inciting playful experiences were also quite diverse, including humor, gameplay, imagination play, work activities, travel and conversations with friends with these different playful memories causing somewhat similar consequential wellbeing effects. These reported wellbeing consequences, while often overlapping, did not always occur in all experiences. This current work is limited in terms of its ability to investigate comparative differences, as its goal is qualitative integration. However, this probably speaks to how diverse forms of play/playfulness, while divisible from each other are also somewhat connected at least from participants’ perspectives. If diverse play behaviors serve a more general playful function in individual’s lives, then it may be essential to study individual play behaviors in context to generalized levels of playful experience (Masek and Stenros, 2024).

For example, in past studies playing video games found a reduction in pro-social behavior correlated with more than 9 h of competitive gaming a week (Lobel et al., 2017), but the same study found no result with fewer hours played or other types of gaming. This mix of results may in part be due to only looking at a small part of the picture of playful behavior in those developing lives. For example, if someone is playing 9 h of games, *replacing* other forms of playful behaviors then it is possible that what was being observed was the effect of video games replacing other non-digital forms of play, such as offline social interaction.

Several of these wellbeing associations align with past empirical findings and theories of playfulness. Positive emotion is aligned with claims that playfulness as a personality trait is fun-seeking (Shen et al., 2014), that playfulness may functionally cause “fun and laughter” (Barabadi et al., 2022) it also aligns with studies that more playful people have higher reported positive emotions in life (Farley et al., 2021; Tonkin and Whitaker, 2019) and playfulness interventions cause increases in positive affect (Proyer et al., 2021). Stress reduction would align with the OLIW’s lighthearted characteristic of “not worrying too much about future consequences of one’s own behavior;” (2017, p. 4) and APTS’s “Uninhibitedness” (Shen et al., 2014) and studies that playfulness is associated as ameliorating depression (Proyer et al., 2021). Stress reduction is also in alignment with two of Proyer (2014)‘s functions for playfulness “coping strategies” and “coping with situations”. Social bonding would align with OLIW’s *other-directed* factor defined as using playfulness “to make social relations more interesting or to loosen up tense situations with others” (Proyer, 2017, p. 4), it aligns with studies on more playful people having higher reported relationship satisfaction (Proyer et al., 2019) and the *Five Ways to Wellbeing*’s “connect” category (Tonkin and Whitaker, 2021). Social Bonding is also reinforcing of Barabadi et al. (2022) playful functions of “cultivating relationships” and Proyer (2014) “relationships”. Finally subjective learning would support Bateson and Martin, 2013 claim that playful play causes “a spontaneous and flexible” (p. 100) way of being or thinking and the *Five Ways to Wellbeing*’s “keep learning” category (Tonkin and Whitaker, 2021). Certain forms of subjective learning also used the language of *creativity* reinforcing both Barabadi et al. (2022) and Proyer (2014), others used the language of playfulness teaching them “hard work” which would seem aligned with the functions of “mastery orientation” (Barabadi et al., 2022; Proyer, 2014). Thusly, past works are generally reinforced by the current study, but there is still a value in a comprehensive presentation for how individuals report these wellbeing effects in their own words from specific lived experiences.

This framework enables a different approach to integrating studies of human wellbeing, as it relates to playful behavior, across ages. Longitudinal studies of wellbeing find results for social connectedness often typified by playing in children (e.g., Olsson et al., 2013) and then for older adolescents and adults shifting to less play-coded language such as “participation in clubs and groups” (Olsson et al., 2013, p. 5). Participants reported their highly playful adult experiences as commonly occurring in clubs and groups (Masek and Stenros, 2024). Thus, there is a possible reality that because playful phenomena is not labeled as playful for older ages, that studies are secretly studying effects of generalized levels of playful experience and its wellbeing consequences, without the proper context to integrate those effects across studies.

### 4.1 Limitations

The methodology underpinning this work has limitations. Individual self-reports are uniquely biased and prone to failures in memory. Participants had an open view between play, playfulness, games, humor, imagination etc. This is an interesting finding in one way but also a limitation if future scholars wish to investigate only one of those ideas.

The ethnographic approach taken in the study, while enabling a diverse data in its goal of cross-cultural playful experiences, is also limited in other important ways, especially in representing playful experience over the age of 30. There are other diverse groups that should be studied in the future. These respondents are not statistically-representative of any single group and there is the possibility that the interviewees who decided to participate were non-representative or structurally biased in some way.

The coding required interpretation by the scholar. Respondents sometimes described wellbeing effects in more ambiguous terms such as “also the bonding stuff” (E39) which could refer to a temporary state or a longer effect from an environment. This kind of need for interpretation is a common element of exploratory and qualitative work (see Glaser and Strauss, 1998), and all utterances and interpretation can be reviewed in table form in Supplementary materials.

## 5 Conclusion

This work has provided the findings of a bottom-up qualitative interview on 125 highly playful experiences from individuals from 42 national backgrounds around the world for how respondents described its “meaning.” The resulting meaningful consequences are analyzed as four components all of which are in alignment with several theories of wellbeing: positive emotion, stress reduction, social bonding, and learning/growth. In addition, these four forms of wellbeing had differing degrees of consequence from a transient state, an environment that reliably caused an effect, to a change in broader individual approaches to life. Whereas past works have usually investigated some subset of these presentations for playful wellbeing, this work presents them as interconnected for individuals. This work proposes the connection is one based on repetition. It hypothesizes that highly playful experiences engender states associated with wellbeing, that when repeated sufficiently become associated to an environment of playfulness and wellbeing, that when repeated sufficiently becomes incorporated into an individual’s broader personality and approach to playfulness and wellbeing in multiple contexts.

This investigation concludes that to study individual playful behavior, whether it be games, play, humor, partying, and even more critically positive relationships such as friendship or playful enjoyment, one should address the context of playfulness writ large. This large-scope of playfulness as all engagement-priority organized phenomena has a historical and contemporary basis for positive psychology. This paper acknowledges and re-opens an under addressed question- if such profound theories as Flow, Self Determination Theory, or Broaden and Build can be based in the diverse starting points of “interest,” “enjoyment,” “puzzles,” “games,” “humor,” “play,” “mountain climbing,” “art” and “creativity”- should we not study these diverse phenomena as key contexts for each other? This paper argues that playfulness is not only important for wellbeing, but also that wellbeing, for individuals, is seen as the primary meaningful consequence of playfulness.

In this way, studying playfulness in adult life should be a primary interest of those who study wellbeing. Considering that participants discussed several off the shelf games in their HPEs, it may be that games as designed objects are fertile territory for positive wellbeing interventions. Subsequent research questions that should be investigated include: do intentional playful experiences inspire positive emotion, a relief of life-stress, bonding for those involved, or learning about one’s subjective-objective position in the world? Other valuable studies would include a more representative study across culture for how playfulness and wellbeing interact, a longitudinal study on how playful behavior changes over individual’s lifespans, and how playfulness operates as an outcome/causal factor for wellbeing. In addition, the opposite relationship should be tested: does wellbeing cause playfulness? In conclusion, highly playful experiences are described as causing meaningful wellbeing in individuals lives from a variety of cultural backgrounds.

## Data Availability

The original contributions presented in the study are included in the article/Supplementary material, further inquiries can be directed to the corresponding author.

## References

[ref1] Apter M. J. (1991). Adult Play: A Reversal Theory Approach. Amsterdam: Swets and Zeitlinger.

[ref2] Apter M. J. (2001). Motivational Styles in Everyday Life. Washington, DC: American Psychological Association.

[ref3] Arrasvuori J. Boberg M. Holopainen J. Korhonen H. Lucero A. (2011). “Applying the PLEX framework in designing for playfulness.” Proceedings of the 2011 conference on designing pleasurable products and interfaces. 1–8.

[ref4] Barabadi E. Elahi Shirvan M. Shahnama M. Proyer R. T. (2022). Perceived functions of playfulness in adult English as a foreign language learners: an exploratory study. Front. Psychol. 12:823123. doi: 10.3389/fpsyg.2021.823123, 35140669 PMC8818999

[ref5] Barnett L. A. (2017). The inculcation of adult playfulness: from west to east. Int. J. Play 6, 255–271. doi: 10.1080/21594937.2017.1383010

[ref6] Bateson P. Martin P. (2013). Play, Playfulness, Creativity, and Innovation. Cambridge, United Kingdom: Cambridge University Press.

[ref8002] Beard K. S. (2015). Theoretically speaking: An interview with Mihaly Csikszentmihalyi on flow theory development and its usefulness in addressing contemporary challenges in education. Edu Psychol Rev. 27, 353–364.

[ref9002] Besio S. Amelina N. (2017). Play in children with physical impairment. Play development in children with disabilities, 120–136.

[ref7] Brauer K. Sendatzki R. Scherrer T. Chick G. Proyer R. T. (2023). Revisiting adult playfulness and relationship satisfaction: APIM analyses of middle-aged and older couples. Int. J. Appl. Posit. Psychol. 8, 227–255. doi: 10.1007/s41042-021-00058-8

[ref8] Brauer K. Stumpf H. S. C. Proyer R. T. (2024). Playfulness in middle- and older age: testing associations with life satisfaction, character strengths, and flourishing. Aging Ment. Health 28, 1540–1549. doi: 10.1080/13607863.2024.2372471, 38940664

[ref9] Brown S. Vaughan C. (2009). Play: How it Shapes the Brain, Opens the Imagination, and Invigorates the Soul. New York: Penguin.

[ref10] Bundy A. C. Nelson L. Metzger M. Bingaman K. (2001). Validity and reliability of a test of playfulness. Occup. Ther. J. Res. 21, 276–292. doi: 10.1177/153944920102100405

[ref11] Burghardt G. M. (2005). The Genesis of Animal Play: Testing the Limits. Cambridge, MA: MIT press.

[ref9012] Csíkszentmihályi M. (1975). Beyond Boredom and Anxiety. San Francisco: Jossey-bass.

[ref12] Chang P. J. Yarnal C. Chick G. (2016). The longitudinal association between playfulness and resilience in older women engaged in the red hat society. J. Leis. Res. 48, 210–227. doi: 10.18666/jlr-2016-v48-i3-6256

[ref13] Charmaz C. (2014). Constructing Grounded Theory. Thousand Oaks: Sage.

[ref15] Deci E. L. Ryan R. M. (2000). The" what" and" why" of goal pursuits: human needs and the self-determination of behavior. Psychol. Inq. 11, 227–268.

[ref16] Deci E. L. Ryan R. M. (2012). Self-determination theory. Handbook Theories Social Psychol. 1, 416–436. doi: 10.4135/9781446249215.n21, 42286773

[ref17] Deterding S. (2016). “Make-believe in gameful and playful design,” in Digital Make-Believe, eds. Turner P. Tuomas Harviainen J. (Cham: Springer International Publishing), 101–124.

[ref18] Deterding S. Dixon D. Khaled R. Nacke L. (2011) “From game design elements to gamefulness: defining" gamification"” *Proceedings of the 15th International academic MindTrek Conference: Envisioning Future media Environments* 9–15

[ref19] Farley A. Kennedy-Behr A. Brown T. (2021). An investigation into the relationship between playfulness and well-being in Australian adults: an exploratory study. OTJR: Occupation, Participation and Health 41, 56–64. doi: 10.1177/1539449220945311, 32723209

[ref20] Fehr K. K. Boog K. E. Leraas B. C. (2019). “Play behaviors: definition and typology,” in The Encyclopedia of child and Adolescent Development, eds. Hupp S. Jewell J. (Hoboken: Wiley), 1–10.

[ref8004] Fielding N. (2008). Ethnography. In Researching social life. Sage. 145–163.

[ref21] Fredrickson B. L. (2004). The broaden–and–build theory of positive emotions. Philos. Trans. R. Soc. Lond. Ser. B Biol. Sci. 359, 1367–1377. doi: 10.1098/rstb.2004.1512, 15347528 PMC1693418

[ref22] Fredrickson B. L. (2013). “Positive emotions broaden and build,” in Advances in Experimental social Psychology, eds. Devine P. Plant A. (San Diego, CA: Academic Press), 47, 1–53.

[ref23] Glaser B. G. Strauss A. L. (1998). Grounded Theory. Strategien Qualitativer Forschung. Bern: Huber, 4.

[ref24] Gordon G. (2014). Well played: the origins and future of playfulness. Am. J. Play 6, 234–266.

[ref25] Gray P. (2011). The decline of play and the rise of psychopathology in children and adolescents. Am. J. Play 3, 443–463.

[ref26] Gross J. J. (2015). Emotion regulation: current status and future prospects. Psychol. Inq. 26, 1–26. doi: 10.1080/1047840x.2014.940781

[ref27] Guitard P. Ferland F. Dutil É. (2005). Toward a better understanding of playfulness in adults. OTJR 25, 9–22. doi: 10.1177/153944920502500103

[ref28] Haig B. D. (2013). Detecting psychological phenomena: taking bottom-up research seriously. Am. J. Psychol. 126, 135–153. doi: 10.5406/amerjpsyc.126.2.0135, 23858950

[ref29] Kallio H. Pietilä A. M. Johnson M. Kangasniemi M. (2016). Systematic methodological review: developing a framework for a qualitative semi-structured interview guide. J. Adv. Nurs. 72, 2954–2965. doi: 10.1111/jan.13031, 27221824

[ref30] Lobel A. Engels R. C. Stone L. L. Burk W. J. Granic I. (2017). Video gaming and children’s psychosocial wellbeing: a longitudinal study. J. Youth Adolesc. 46, 884–897. doi: 10.1007/s10964-017-0646-z, 28224404 PMC5346125

[ref31] Lubbers K. Cadwallader J. Lin Q. Clifford C. Frazier L. D. (2023). Adult play and playfulness: a qualitative exploration of its meanings and importance. J. Play Adulthood 5, 1–19. doi: 10.5920/jpa.1258

[ref32] Magnuson C. D. Barnett L. A. (2013). The playful advantage: how playfulness enhances coping with stress. Leis. Sci. 35, 129–144. doi: 10.1080/01490400.2013.761905

[ref33] Masek L. (2023a). “Criticizing Caillois: examining how players perceive rules in play and games.” In *Conference Proceedings of DiGRA 2023 Conference: Limits and Margins of Games Settings*.

[ref34] Masek L. (2023b). “How playfulness can enable greater understanding of game-based adult mental health interventions.” In *International Simulation and Gaming Association Conference* (pp. 171–184). Springer Cham

[ref35] Masek L. (2025). Identifying playfulness: an empirical study on how adults recognize and define playfulness across culture. Games Cult. 20, 856–877. doi: 10.1177/15554120231226262

[ref9006] Masek L. Stenros J. (2021). The meaning of playfulness: a review of the contemporary definitions of the concept across disciplines. Eludamos: Journal for Computer Game Culture 12, 13–37. doi: 10.7557/23.6361, 42355355

[ref9005] Masek L. Stenros J. (2024). Parties as playful experiences: why game studies should study partying. Eludamos: Journal for Computer Game Culture 15, 75–96. doi: 10.7557/23.7562, 42343451

[ref36] Maslow A. H. (1971a). The farther Reaches of human Nature, vol. 1971 New York: Viking Press.

[ref37] Maslow A. H. (1971b). Peak experiences in education and art. Theory Pract. 10, 149–153.

[ref38] McDonald M. G. Wearing S. Ponting J. (2009). The nature of peak experience in wilderness. Humanist. Psychol. 37, 370–385. doi: 10.1080/08873260701828912

[ref39] Mouton A. R. Montijo M. N. (2017). Love, passion, and peak experience: a qualitative study on six continents. J. Posit. Psychol. 12, 263–280. doi: 10.1080/17439760.2016.1225117

[ref40] Oades L. G. Mossman L. (2017). The Science of Wellbeing and Positive Psychology.

[ref41] Olsson C. A. McGee R. Nada-Raja S. Williams S. M. (2013). A 32-year longitudinal study of child and adolescent pathways to well-being in adulthood. J. Happiness Stud. 14, 1069–1083. doi: 10.1007/s10902-012-9369-8

[ref42] Parker C. Kennedy-Behr A. Wright S. Brown T. (2023). Does the self-reported playfulness of older adults influence their wellbeing? An exploratory study. Scand. J. Occup. Ther. 30, 86–97. doi: 10.1080/11038128.2022.2145993, 36409561

[ref8001] Pearton J. L. Ramugondo E. Cloete L. Cordier R. (2014). Playfulness and prenatal alcohol exposure: a comparative study. Australian Occup Therapy J. 61, 259–267. 10.1111/1440-1630.12118 24547920

[ref43] Petitmengin C. (2006). Describing one’s subjective experience in the second person: an interview method for the science of consciousness. Phenomenol. Cogn. Sci. 5, 229–269. doi: 10.1007/s11097-006-9022-2

[ref44] Proyer R. T. (2012). Development and initial assessment of a short measure for adult playfulness: the SMAP. Personal. Individ. Differ. 53, 989–994. doi: 10.1016/j.paid.2012.07.018

[ref45] Proyer R. T. (2014). Perceived functions of playfulness in adults: does it mobilize you at work, rest, and when being with others? Eur. Rev. Appl. Psychol. 64, 241–250. doi: 10.1016/j.erap.2014.06.001

[ref46] Proyer R. T. (2017). A new structural model for the study of adult playfulness: assessment and exploration of an understudied individual differences variable. Personal. Individ. Differ. 108, 113–122. doi: 10.1016/j.paid.2016.12.011

[ref47] Proyer R. T. (2018). Playfulness and humor in psychology: an overview and update. Humor 31, 259–271. doi: 10.1515/humor-2016-0080

[ref48] Proyer R. T. Brauer K. Wolf A. Chick G. (2019). Adult playfulness and relationship satisfaction: an APIM analysis of romantic couples. J. Res. Pers. 79, 40–48. doi: 10.1016/j.jrp.2019.02.001

[ref49] Proyer R. T. Gander F. Bertenshaw E. J. Brauer K. (2018). The positive relationships of playfulness with indicators of health, activity, and physical fitness. Front. Psychol. 9:325276. doi: 10.3389/fpsyg.2018.01440, 30154749 PMC6102740

[ref50] Proyer R. T. Gander F. Brauer K. Chick G. (2021). Can playfulness be stimulated? A randomised placebo-controlled online playfulness intervention study on effects on trait playfulness, well-being, and depression. Appl. Psychol. Health Well Being 13, 129–151. doi: 10.1111/aphw.12220, 32844597

[ref53] Schechner R. (2017). Performance Studies: An Introduction. New York: Routledge.

[ref54] Scott D. G. Evans J. (2010). Peak experience project. Int. J. Child. Spirit. 15, 143–158. doi: 10.1080/1364436x.2010.497648

[ref55] Seligman M. E. (2011). Flourish: A Visionary new Understanding of Happiness and well-Being. New York: Simon and Schuster.

[ref56] Shen X. (2020). Constructing an interactionist framework for playfulness research: adding psychological situations and playful states. J. Leis. Res. 51, 536–558. doi: 10.1080/00222216.2020.1748551

[ref57] Shen X. S. Chick G. Zinn H. (2014). Validating the adult playfulness trait scale (APTS): an examination of personality, behavior, attitude, and perception in the nomological network of playfulness. Am. J. Play 6, 345–369.

[ref59] Shen X. Liu H. Song R. (2021). Toward a culture-sensitive approach to playfulness research: development of the adult playfulness trait scale-Chinese version and an alternative measurement model. J. Leis. Res. 52, 401–423. doi: 10.1080/00222216.2020.1850193

[ref60] Shen X. Masek L. (2024). The playful mediator, moderator, or outcome? An integrative review of the roles of play and playfulness in adult-centered psychological interventions for mental health. J. Posit. Psychol. 19, 1037–1050. doi: 10.1080/17439760.2023.2288955, 37339054

[ref63] Skaggs P. Twede B. (2023). Play and creativity. J. Higher Educ. Theory Practice 23, 167–173. doi: 10.33423/jhetp.v23i11.6227, 42266785

[ref66] Staempfli M. B. (2007). Adolescent playfulness, stress perception, coping and well being. J. Leis. Res. 39, 393–412. doi: 10.1080/00222216.2007.11950114

[ref14] Strauss A. L. Corbin J. M. (1997). Grounded Theory in Practice. Thousand Oaks: Sage.

[ref68] Suri H. (2011). Purposeful sampling in qualitative research synthesis. Qual. Res. J. 11, 63–75. doi: 10.3316/qrj1102063

[ref8003] Thurmond V. A. (2001). The point of triangulation. J Nur Scholarship 33, 253–258. 10.1111/j.1547-5069.2001.00253.x 11552552

[ref69] Teppo A. R. (2014). “Grounded theory methods,” in Approaches to Qualitative Research in Mathematics Education: Examples of Methodology and Methods, eds. Bikner-Ahsbahs A. Knipping C. Presmeg N. (Dordrecht: Springer Netherlands), 3–21.

[ref70] Tonkin A. Whitaker J. (2019). Play and Playfulness for Public Health and Wellbeing. Oxon: Routledge.

[ref71] Tonkin A. Whitaker J. (2021). Play and playfulness for health and wellbeing: a panacea for mitigating the impact of coronavirus (COVID 19). Soc. Sci. Human. Open 4:100142. doi: 10.1016/j.ssaho.2021.100142, 34927056 PMC8665352

[ref72] Trammell A. (2020). Torture, play, and the black experience. G| A| M| E Games as Art, Media, Entertainment 1, 33–49.

[ref73] Vahlo J. Tuuri K. Välisalo T. (2022). Exploring gameful motivation of autonomous learners. Front. Psychol. 13:825840. doi: 10.3389/fpsyg.2022.825840, 35282190 PMC8916124

[ref74] Webster J. Martocchio J. J. (1992). Microcomputer playfulness: development of a measure with workplace implications. MIS Q. 16, 201–226.

[ref75] Winnicott D. W. (1971). Playing and Reality. East Sussex: Psychology Press.

[ref76] Yin R. K. (2015). Qualitative Research from start to Finish. New York: Guilford publications.

