# Supplementary material for: Playfulness causes meaningful wellbeing: an investigation of 125 global playful memories
Source: Front Psychol. 2026 Jul 8;17:1818085. doi: 10.3389/fpsyg.2026.1818085 (PMC13388034; doi:10.3389/fpsyg.2026.1818085)
Supplement: Supplementary file 1 [file Table_1.DOCX]

| Theme | Quote |
| --- | --- |
| State Social Bonding | I mentioned the sort of unity, the sort of hive mind kind of you just are one with the audience and then and I suppose part of that is also like connecting with people. Maybe just like briefly and just mostly just physically you can't necessarily talk much there of course, given the nature of situation but maybe I don't know because this was a brief moment of impromptu fun with some person I did not know beforehand at all right. And it was. Yeah, fulfilling, maybe like, not just in the context of what happens immediately then. Yeah, but maybe like, in what it symbolizes, as in the way that I have the experience that this is a space and a situation where people are open and playful enough to do something like that with random people without any like, consideration. Short sort of light atmosphere of friendliness (E3) |
| State Social Bonding | I remember feeling such an innate sense of wholeness, and like fitting in, I guess. And just like camaraderie, and having fun and not caring, and all boundaries and walls, we're just kind of let down and everyone was on the same playing field. And we're just having fun. So we're laughing like crazy. .... I remember feeling very light. I remember not feeling guarded at all. I felt very. I wasn't comparing myself to anyone, which is something I think a lot of people in high school do. It's all about who looks like this, or how am I presenting, but it was everyone was just, you didn't care. Like you were? I don't know. Everyone was like the same person, if that makes sense. It was just I wasn't thinking about, Oh, am I messing up my lines? Because I was messing up their lines. I'm not thinking about how does my hair look? Am I like acting weird? Am I being too much? Because everyone was being too much or doing too weird. So it was just like a very physical sense of like, completeness, and no anxiety, no worry. Just kind of like energetic feeling, you know, like, very vibrant, (E8) |
| State Social Bonding | The happiness and also like feeling close to my opponent and my partner. it's playfulness. And even with my my opponents, yes, the same for like we were all sharing this moment. And like, but we didn't talk about it. So it was like it was in my mind that I was really happy to feel this playfulness during the whole game. And we lost at the end of it. I mean, it was one of my best memory of activities. (E10) |
| State Social Bonding | it's a feeling of community and sharing. (E13) |
| State Social Bonding | I guess, long term, technically not much. But like, in the moment, it was like an enjoyable, like fun time to spend with people that I liked (E40) |
| State Social Bonding | the funny parts, but I would say it was really a way for me to get closer to my family and cooperate when in a time when we didn't have a lot of dialogue. So it is kind of it's not the main thing that got us closer, but it helped. So it's one of the many factors that could help you (E93) |
| State Social Bonding | We there's something we can do and We can cooperate with each other and we are not alone like before that we feel lonely because we just do what they want to do and we don't make much friends (E94) |
| State Social Bonding | And there were there were times when I didn't win as well, I lost and I felt like I let people down. But it was a part of the experience as well. And I heard good things from my like, you know, really touching things. Like I said earlier, what when I teacher told me that, that I wanted as hard as our class with my classmates as well, they weren't bummed that I lost or something. But they were supporting me. And they were just striving me to go forward, striving me to do better. That whole experience is pretty important, and also playful in the same way. (E97) |
| Environment Social Bonding | Now that we are a family with our dog, sometimes we include him in the, in the playfulness, and we remember and cherish those moments as well. So yeah, it's all about house building, I guess, home building. house building. (E125) |
| Environment of Social Bonding | it just feels so like so hard when you enter this kind of like huge environment and you get like, rejected and you can't make friends and you don't know how things go around. So It felt like a big deal to not make people feel rejected even though if they didn't want to participate, like to show that they were welcome anyway, if they wanted to be. (E1) |
| Environment of Social Bonding | the game makes your friends closer to you (E15) |
| Environment of Social Bonding | I think that actually, like really built our friendship up to that's it's still today, like we, we just cause shenanigans. We just do fun, silly stuff. Yeah. We're not like really close, personal friends who talk about deep things, but we always get together to do dumb stuff. (E17) |
| Environment of Social Bonding | I would say what is fun is like, the interactions with my family (E18) |
| Environment of Social Bonding | I think in addition to those, it's also for me, it's sort of, like, one of these, like community moments and and having, having, like, strong friendships and, and people who, who are supportive in this sort of like, so that they sort of they are having fun with you. (E22) |
| Environment of Social Bonding | It's not a super important meaning. But it's Well, yes, it is important, because it's like the time we spend with my friends playing, I really enjoy. And it's like, it's the little moment of the week or time when I play that is like I there's not kind of obligations that small university or projects or work or problem is that like, two hours that we're playing and having fun. (E25) |
| Environment of Social Bonding | Yeah, it goes again, with relationship with my brothers, even with our family in general, like, or we've gotten our parents to play. We play with our close friends, like often. Like I, I came here to a different country, and I still got the table because I still want to play and teach everyone how to play. Because Yeah, just like, it's, it's one of my favorite games. And it's just an opportunity to be playful, and to have fun. And that goes back to, like, my ideal day is just spending time with people that I love, and just having, like a fun game with them. And this is a way that I can do that. (E32) |
| Environment of Social Bonding | I'm thinking that this relationship is maybe the most playful thing, the way we interacted ... quite possibly the most important thing, The best and most rewarding thing that has happened. (E33) |
| Environment of Social Bonding | It's a little thing that I do that we do between friends. That makes us laugh (E35) |
| Environment of Social Bonding | I'd say the bonding is the most important part. Yeah. Like I feel more connected to these friends who might wrestled with, it's, it was definitely that. Or like, the sort of biggest thing about it. I guess the other thing that was really important to me, was that like, how to put it? I guess in some ways, it taught me also to be a sort of a little bit more spontaneous in this sense. (E38) |
| Environment of Social Bonding | I think it's just like, it's also the bonding stuff that I mentioned in previous thing, (E39) |
| Environment of Social Bonding | Well, I think it's, to me, it's been a little bit about kind of expressing myself and like the my relationship with nature, like, being friends with the, you know, and in terms with the nature and kind of using what's in the nature to inspire myself, rather than just like, I don't know, destroying it. So kind of like, using what is in the nature to, you know, bring myself joy. So I feel I kind of like, made my relationship closer with that certain forest in a way, because like, I became more familiar with it. And I had these kind of, like, you know, fun, playful moments in it. So I got a flack, you know, got more attached to it in a way I feel. (E42) |
| Environment of Social Bonding | Yeah, I feel like from my relationship, I mean, I think it's important that you share stuff. I mean, everyone, that you share stuff with the person you love, or anything with your friends or with your, your brother. So I think that's just like adding a new thing that I can share with. with people, I mean, my boyfriend and my brother, so like, with people I love. So I mean, I think the more you have experiences like that, in which you're able to share a moment and like, the degree, the best it is. (E43) |
| Environment of Social Bonding | I think it gave me a very strong sense of safety and belonging. I found it to be a very kind of comforting presence. I found it to be a very I guess, you know, safe presence in my life, especially at that age and that stage in my life. (E47) |
| Environment of Social Bonding | it's really, really giving, making good memory with friends. And sometimes. I sometimes think, oh, there was a really good moment with her. So yeah, we have to go load about songs together. Like later, I sometimes text like that. So it's, yeah, it's like giving me good memory and make me to go there again with my friends. (E55) |
| Environment of Social Bonding | and there's a sense of camaraderie, because like, yes, also that role for you. Yeah, I think that does meaning I think it's, it's interesting as well for me, because it's cuz we both can get into this state and just play like there's. And [Room mate] is very quiet and usually very close up and stuff. So that's, that's pretty cool. (E57) |
| Environment of Social Bonding | I think for me, that was for me, let's say a starting point because after that, we became closer. (E61) |
| Environment of Social Bonding | It can be put in different ways. But the best way I would like to put is like giving back to community. I think it gives a feeling of being part of community. So you feel responsible for giving back somehow? Well, I might have been too much deep about it. But I think that's how it has affected my personal life by festivals. It's always a [religious] community, and you'd like to celebrate with the community or you want to be the part that invites the singer and everyone in your house and you would want to get those food and everything. So we are giving back to the community, I think that can be taken in different ways. But leave link with this festival. It will be food money or whatever, donations or whatever. But it's like giving back to communities. The value that came in. [E63] |
| Environment of Social Bonding | I think it helped cement friendship, and deepen our friendship. I'm still friends with all these people. And I mean, we talk about and reminisce about the things that we used to do all the time...And so yeah, being able to see them in different situations, and respond in those ways, allowed me to know them better, and allowed us to talk about things that maybe we wouldn't normally talk about. And so it allows you to prove that relationship a little bit more and, and have more meaningful conversation. (E66) |
| Environment of Social Bonding | We are like, really friends. Because we had taken the risk together. (E69) |
| Environment of Social Bonding | It's been quite comforting. I guess that's also a little bit about the relationship I have with my friends that specifically this friend. And as I mentioned, it's always been something that we could just kind of go back like when I was in high school. (E76) |
| Environment of Social Bonding | Oh, the meaning. The meaning. To me, it imbues the community, brings togetherness. People no longer see themselves as fractions, but as a whole, like part of something. The problem with adults is the fact that when you see someone, you class the person. Subconsciously, most of the things, we don't do it from our consciousness. We're just like, oh, yeah, this is the guy. Then we bring in this adult problem of racism, class, segmentation, and the rest of them. But the children don't see colors. They don't see those things. They don't see, they don't have those kind of wirings we have in us. They don't have it in them yet. So doing that makes the adults feel free to interact with themselves. I mean, what's the point when you're playing a game with another dad, and you fall? Everyone has seen you fall, so just get up, man, and everyone feels free to interact. So it brings togetherness. To me, that's just the meaning.... People feel free to interact with themselves to an extent. (E77) |
| Environment of Social Bonding | I understood that the parents are the ones who really support us, who are the main people that we maybe should care about. And it's not that quickly that you can add another person to your life and make it... You say significant other for the husband and wife, but here we can say significant other as another person, a person of your life. It might be friends, family members and stuff. So it's too difficult to find another person who will care about you....Because even in that, as I said before, I was afraid to disappoint them, but they were supportive, maybe, I don't know. And I saw that there is such a thing, if you do not care about yourself, nobody will. And I just saw that it's not true that much. We have also family members and they will care about you to some level. (E78) |
| Environment of Social Bonding | I don't know. I guess it just brought my cousins and people, all of us together quite a bit. And yeah, so we still even though there's a lot of us and since people are married now, there's twice as much. So there's a lot of us, but I would say we are still pretty close. And like I said, if we get together, then it's always still just like it was back in the day. So I think that's what it means to me, just like the people and the experiences. (E84) |
| Environment of Social Bonding | I think I just got close to my friends. Really. I mean, I'm still like I said, I'm still talking to these girls. Very close. We obviously are in different parts of the world at this point. But yeah, so I don't know when all of us will even ever be together again, to be honest, but yeah, I mean, it's nice to know that there's people like that. So yeah. (E85) |
| Environment of Social Bonding | I think it has a huge meaning right now. Because I'm new here. And those are like my closest friend at in Finland. So it really helps me. Like getting through our days without any, you know, other friends. So that's that that's meaningful. That has a lot of meaning to me. Yeah (E88) |
| Environment of Social Bonding | And I made a lot of friends. I got to know a lot of people. I got to play with somebody I looked up to think these are all the things I've said earlier. (E98) |
| Environment of Social Bonding | It created a very good bond among three of us, and many now whenever we meet, we get you know, we, we look back into our memories. (E101) |
| Environment of Social Bonding | And, and really like I feel like it was I also met a boyfriend. I mean, like, my boyfriend for the next four years. I met him there. So it was a really, really important experience that trip. (E115) |
| Environment of Social Bonding | I understood that the parents are the ones who really support us, who are the main people that we maybe should care about. And it's not that quickly that you can add another person to your life and make it... You say significant other for the husband and wife, but here we can say significant other as another person, a person of your life. It might be friends, family members and stuff. So it's too difficult to find another person who will care about you....Because even in that, as I said before, I was afraid to disappoint them, but they were supportive, maybe, I don't know. And I saw that there is such a thing, if you do not care about yourself, nobody will. And I just saw that it's not true that much. We have also family members and they will care about you to some level. (E78) |
| Environment of Social Bonding | It kind of is included in the like [Holiday] and New Year coming. And like this transition from this year to next year. The transition the whole thing from about, let's say 20th of December to about first, first of January, this transition period, where you have all your family time. From the time this experience new year. Kind of sort of thinking about the next year, it's kind of like, it's sort of light zeroing of how would I say it? I don't know how to say it, but like, wrapping up, the current year, that whole experience that time window after [Holiday] and New Year, kinda like belongs into that package. Yes.Yeah, yeah. It belongs it's not the same gravity as [Holiday] time with your family, but it's on the same bucket kind of things. Yes, it belongs to the period and without it, it's kinda like something is missing. (E83) |
| Personality Shift of Social Bonding | Yeah, it's just that kind of that situation and me realizing how much fun it is. influences the experiences that I seek out now in everyday life. So if I like any situation, I want to just have fun, close friends and have a good time with them. Like that's, that's what matters. So like, I just sit down and play a game with friends, like card game, or anything. That's always way more fun than just like, going out to some loud club or whatever. Just doing something else. Like I'd rather just find my people, my community, and just have fun with them." (E31) |
| Personality Shift of Social Bonding | In my life, maybe I feel more confident. Because actually, I'm a bit shy person. Really hard to get good the other friends was there I was so brave to talk to others people and talk to stranger and make friends and heard about others like kind of cultural nomads. So in my life, I felt more confident and more outgoing person I think because I'm usually just playing games, where I know I felt It is okay to go outside and play with other people. (E50) |
| Personality Shift of Social Bonding | You know, the meaning is that, in my life, I try to give more space like more ideas or concepts to my friendship for people. And the moment I talk about my childhood experience, I always remember those guys, my friends, and which means it created a certain kind of good impression, how can I be with friend to create that relaxation, like that kind of moments together? And how life would be so interesting when you have people together, that can understand each other. So the friendship meaning after that one is huge. Because one of my friends is still we’re friends and Even when I just introduce him to other people, I don't want to call him my friend. I just tell them He's my my brother. Because since then, we are just friends. And even I just give him my account, my all the names all this access numbers of my account, because I trust him. So this trust comes because we have been together for a long period of time playing together, having fun together and studying in the same room. And that creates a strong attachment. So yeah, this is very important and that leaves in my, in my mind always. (E59) |
| Personality Shift of Social Bonding | One thing, That’s the turning point, I tried to talk to people more. In my, like my elementary school, I was so introverted, but still, I couldn't talk after the football match and something. But in my highschool, I have those friends and those friends have so many friends, other friends and classmates. So the moment I am with some, I'm always communicating with others as well. Not only in that way, we have been fortunate enough with this guy, who I call, my brother, we went to the same university and the same campus. And because he knows me, well, in my high school, when we are together with people, he tries to always introduce me and tells about me, my character, “like, if you meet him on your way, he might not first say hi, you should say him Hi. He tries to like yeah, you know, he convinced people and he showed me He taught me lessons. And so being in that group itself helps me be socially. (E60) |
| Personality Shift of Social Bonding | And also shaped like me to be like, playful in general? Like, I like to socialize, maybe because I play a lot as well. [E64] |
| Personality Shift of Social Bonding | it's valuable for me. Because I suppose I love spending time with people. Especially I love being there. Like, in a home circumstance, like, somewhere, somewhere which is comfortable for me to feel like home. And also, I love getting know, getting to know people. I love hearing their own stories and feelings. And I love people. (E70) |
| Personality Shift of Social Bonding | I became a little bit more friendlier, a bit more social. And then I got a little less I don't know how to explain this, like little less scared of boys maybe. Basically, I became more socially adept. (E71) |
| Personality Shift of Social Bonding | the importance of a work ethic and friendships mostly. So what goals Yeah, I'll just say those two the work ethic and the friendships. (E73) |
| Personality Shift of Social Bonding | This was the first experience for me to go outside my schedule. And it always has an impact, because I told you these trials. This is I remember this vividly because of myself alone. And after that. After that I we had similar experiences, again and again at different places. So and because now he's my fiance, I can't I can't stop it from having these kinds of experiences. So this is why now that experiences has a trickle down effect like a domino effect. Then we went for other things again, and again and again and again. And now have been doing it. Now I have to do it. So right this experience is a base for me, where we started. We started as a couple and slowly transition into and slowly incorporating those kinds of experiences in my life. Now I have an experience that more often than I experienced it earlier. So this experience was a day for me to start with. (E100) |
| Personality Shift of Social Bonding | I hang out a lot with my friends. So even though, you know, I have, just to give you an example, three or four nights, I am not doing my dinner right here in a hostel that I am supposed to have, but I'm just hanging out in the side and eating, you know, having dinners. So, yeah, I think I keep on repeating in some forms just hanging out with my friends. And yeah, I keep on repeating in some form or another (E106) |
| Personality Shift of Social Bonding | It, to be honest with you, these experiences signify friendship for me. I think, like, that's, these experiences and, like, this friend circle taught me how... This is my definition of ideal friendship, like, how you should be with someone. Like, if you're calling somebody your friend, this is how it should be. So you would definitely, like, see me refer to someone as my colleague or classmate. But I wouldn't, like, I wouldn't use the term friend so lightly. Because, like, for me, a friend is, like, someone who...If you, like, go to the surface, go to that certain person, and if I express my feelings to them, they would not judge me in any way, maybe. But they would definitely call me out if I'm doing something wrong. And, like, that's, that's, and, like, they would, like, completely keep your, keep, like, they would, like, not tell somebody that I told them. Like, they wouldn't be, basically, be a snitch. Snitch, or, like, a gossip. Like, they wouldn't gossip about it and stuff like that. So, I think that's, that, I think this, it means friendship for me. (E109) |
| Personality Shift of Social Bonding | Yeah, it's a pretty pretty impactful from the point of view of it's how I socialize with one of my friends, I guess. And it's how I probably perceive I add value in some ways, like social group and everything else. (E116) |
| Personality Shift of Social Bonding | One thing, That’s the turning point, I tried to talk to people more. In my, like my elementary school, I was so introverted, but still, I couldn't talk after the football match and something. But in my highschool, I have those friends and those friends have so many friends, other friends and classmates. So the moment I am with some, I'm always communicating with others as well. Not only in that way, we have been fortunate enough with this guy, who I call, my brother, we went to the same university and the same campus. And because he knows me, well, in my high school, when we are together with people, he tries to always introduce me and tells about me, my character, “like, if you meet him on your way, he might not first say hi, you should say him Hi. He tries to like yeah, you know, he convinced people and he showed me He taught me lessons. And so being in that group itself helps me be socially. (E60) |
| Personality Shift of Social Bonding | I came out of my shell, as I told you, like, I became a little like, a little bit more adventurous. I like learn how to like talk to people, like you know, just need to just go and ask their name and not that I do. But I learned that it's it's the only thing that takes to make friends (E71) |
| Personality Shift of Social Bonding | the meaning that I could extract from this is that communication skill is very important, or, and the socializing skill, I don't know if this can be considered like social engineering or whatever term really fits into this. But the idea is that you know how to deal with a social situation, this was a social situation, you know, we were socializing. But it was getting serious because they were talking about a very important topic. And so the fact that I was able to use, as I said before, some of my soft skills to try to, you know, see where I can go with that. And I think if I really extract this lesson, or if I take the lesson out of this, then it would be like how I use my communication skills, my social, you know, interaction skills, or whatever, is the best time for that. And if I can use it elsewhere in any other social, you know, situation. So I think that's very important. If I can, if I can use that in the rest of my life. (E110) [social skills] |
| State of Positive Emotion | when I think about like, why it was playful and why I enjoyed it was because it was like one of the first times One of the few times I had gotten out of my comfort zone, I was like, I'm going to do this with my friend because we're having fun and I want to keep having fun....I guess, like, pure child's like, or pure childness. Like, well, those moments where you think of kids, they're like, playing this what they do they play, not mindlessly, but they play in a way where nothing really matters. They are like, there's them their friends and fun. That's like they don't really there's like the You might consider consequences. But for the most part, they probably don't. They're just like, I'm going to do this because I want to have fun. And because my friends are doing it, and they want to have fun, so we're all going to do this. So that was like one of those moments, one of those pure, like, childlike moments that I've had of me like being happy having fun with my friend. And it's just also like, that unique experience that sort of makes it stand out. (E4) |
| State of Positive Emotion | specific vibe on this current events when everybody's just just happy and excited about it's happening. And it's very contagious. Okay, you just can't stopped smiling (E5) |
| State of Positive Emotion | I remember feeling such an innate sense of wholeness, and like fitting in, I guess. And just like camaraderie, and having fun and not caring, and all boundaries and walls, we're just kind of let down and everyone was on the same playing field. And we're just having fun. So we're laughing like crazy. .... I remember feeling very light. I remember not feeling guarded at all. I felt very. I wasn't comparing myself to anyone, which is something I think a lot of people in high school do. It's all about who looks like this, or how am I presenting, but it was everyone was just, you didn't care. Like you were? I don't know. Everyone was like the same person, if that makes sense. It was just I wasn't thinking about, Oh, am I messing up my lines? Because I was messing up their lines. I'm not thinking about how does my hair look? Am I like acting weird? Am I being too much? Because everyone was being too much or doing too weird. So it was just like a very physical sense of like, completeness, and no anxiety, no worry. Just kind of like energetic feeling, you know, like, very vibrant, (E8) |
| State of Positive Emotion | it was the best. It was really fun. (E9) |
| State of Positive Emotion | The happiness and also like feeling close to my opponent and my partner. it's playfulness. And even with my my opponents, yes, the same for like we were all sharing this moment. And like, but we didn't talk about it. So it was like it was in my mind that I was really happy to feel this playfulness during the whole game. And we lost at the end of it. I mean, it was one of my best memory of activities. (E10) |
| State of Positive Emotion | the feeling if you feel so happy that you feel that you could be flying (E13) |
| State of Positive Emotion | didn't have a lot of meaning for me... just a fun thing that happens (E19) |
| State of Positive Emotion | So doesn't really have a meaning for me. Just the fun experience. (E20) |
| State of Positive Emotion | It's a very hazy memory... It's fun (E30) |
| State of Positive Emotion | I guess, long term, technically not much. But like, in the moment, it was like an enjoyable, like fun time to spend with people that I liked (E40) |
| State of Positive Emotion | it was a good experience, in that I got to see something that I knew I would completely enjoy. Due to just how over the top and bizarre it is, I guess, I don't know if that's a meaning per se, but yeah. (E41) |
| State of Positive Emotion | Okay, I just found it entertaining. (E45) |
| State of Positive Emotion | I didn't get a lot of comments [on social media], like going to comment, but I got a lot of likes, that I still felt that the response was positive to me. Because like, usually, like To be honest, it's like most of photos have 20 or 30 likes, and that's for the photo just got almost 100 likes there. So I think it was it really contributed to the positive feeling to my action, getting that positive feedback. (E49) |
| State of Positive Emotion | it's just kind of like very good memory, like, you know, kind of, like part of this story and to remember it is very nice, (E53) |
| State of Positive Emotion | Meaning is just happiness. You know, whenever still. Just imagine, still, when, for example, I and my brother talked about it, for example, he played 10 years before, is still we remind some memories and, we laugh, you know, it's some just happiness, nothing more. (E54) |
| State of Positive Emotion | it's really, really giving, making good memory with friends. And sometimes. I sometimes think, oh, there was a really good moment with her. So yeah, we have to go load about songs together. Like later, I sometimes text like that. So it's, yeah, it's like giving me good memory and make me to go there again with my friends. (E55) |
| State of Positive Emotion | I don't think they are, like incredibly meaningful, because they're so short. But I think when I think about things again, that is, it is fun. (E57) |
| State of Positive Emotion | And there were there were times when I didn't win as well, I lost and I felt like I let people down. But it was a part of the experience as well. And I heard good things from my like, you know, really touching things. (E97) |
| State of Positive Emotion | I mean, I can look back to it and then be happy I was it's just a time that I was really enjoying. (E98) |
| State of Positive Emotion | I mean, it sounds like it's not really a big deal. But for me at that point, it was kind of a big deal. Because I felt really like skillful, and I felt like I was in my own domain. And I kind of felt somewhat powerful over this like, academic tsunami, that was like coming to towards me.... So yeah, a combination between like the academic part (E75) [Confidence] |
| Environment of Positive Emotion | Yes, the game itself was just extremely fun. And it was something that I remember so much as a highlight. Like, I could say, four or five experiences with that group of people. But this is one that I that I definitely remember. But yeah, being a kid, I just did random shit. And it felt good. But I don't think it changed me. consciously, maybe subconsciously (E2) |
| Environment of Positive Emotion | Yes, it think it was one of most the best experience I had when. Yeah, definitely. No, because, you know, I'm not. I'm not living in the same country as [country of interview] which has nature everywhere. I'm living next to woods, of course, but I'm not so used to go into the woods, you know, you have woods, but it's not like oh, yeah, I'm going to the woods. Yeah. And so, in this kind of context nowadays, I'm going to do this kind of stuff regularly because of the scouts. But before that I was not. Yeah, so it was something really unusual. Really. And I really liked that.... And doing games because I don't really like to walk for just walking. I prefer to do something nice with people. More than sports in itself, I don’t like sports, but I can easily just go and run and do sports if I’m with people and can have a game experience.... It’s not so much the woods, but nature. and I think this place is really amazing. It's, it's like you have the woods and you have also like, just like land with grass. Yeah. And there's the castle here. So it's, it's a really good place nice place was sunny and you know good weather. Yeah. So it's just like very good and close to home in the same time. So it's like something reachable. And in the center and unusual, I think it was really nice because usually something extraordinary is something totally different very hard. And this was in the same time very simple. But very exciting. (E6) |
| Environment of Positive Emotion | If We happened to go out or to the park or something. And there was like some real reality game organized. I also enjoyed it very much, because it was like, real life inclusion. And you could feel that. I don't know, it's very interesting to have some task completed, for example, with your team or individually, also this feeling of risk, some risk, for example, if you have to catch someone or someone have to catch you, but your aim is not to the caught I think that is the most this comes to my mind in talking about playfulness.... After these transition, I became less flexible, maybe. I mean, like, I was so complicated, have to do different things. And I think that the vector, the way of getting enjoyment pleasure and excitement (E14) |
| Environment of Positive Emotion | it was still really fun. And we did it all the time (E16) |
| Environment of Positive Emotion | It's not a super important meaning. But it's Well, yes, it is important, because it's like the time we spend with my friends playing, I really enjoy. And it's like, it's the little moment of the week or time when I play that is like I there's not kind of obligations that small university or projects or work or problem is that like, two hours that we're playing and having fun. (E25) |
| Environment of Positive Emotion | It honestly made me realize that to talk to virtual characters, like their normal, like their regular people, I was like, I enjoyed that. I enjoy that. I enjoy pretending even though I know, it's just a game, I enjoyed pretending that it was more than it was. And I started kind of actively now looking for opportunities to sneak in some little, you know, bits of participation like that in there.... it changed a little bit, the way that I play the game. And I guess by extension, maybe other games (E27) |
| Environment of Positive Emotion | Yeah, it goes again, with relationship with my brothers, even with our family in general, like, or we've gotten our parents to play. We play with our close friends, like often. Like I, I came here to a different country, and I still got the table because I still want to play and teach everyone how to play. Because Yeah, just like, it's, it's one of my favorite games. And it's just an opportunity to be playful, and to have fun. And that goes back to, like, my ideal day is just spending time with people that I love, and just having, like a fun game with them. And this is a way that I can do that. (E32) |
| Environment of Positive Emotion | It's a little thing that I do that we do between friends. That makes us laugh (E35) |
| Environment of Positive Emotion | It was quite profound. I want to slowly to get back into this kind of activity. But he also more than that, perhaps kind of reminds me of the baseline or that like maximum level? It gave me this idea of what is what is the maximum level and maximum enjoying experience without a cost? Because that's also part of it, maybe I could, I don't know, maybe, maybe I could do drugs and be more enjoy more. But But this has no price. So other than like, any other activity aside your life's economy, but it hasn't. It's not, there's no kind of hidden agendas, or there is no, nothing to be worried about it. (E37) |
| Environment of Positive Emotion | Well, I think it's, to me, it's been a little bit about kind of expressing myself and like the my relationship with nature, like, being friends with the, you know, and in terms with the nature and kind of using what's in the nature to inspire myself, rather than just like, I don't know, destroying it. So kind of like, using what is in the nature to, you know, bring myself joy. So I feel I kind of like, made my relationship closer with that certain forest in a way, because like, I became more familiar with it. And I had these kind of, like, you know, fun, playful moments in it. So I got a flack, you know, got more attached to it in a way I feel. (E42) |
| Environment of Positive Emotion | The meaning it's kind of hard question, but a good one. Well, it's like, rewarding thing I guess. Like when you are completing the completing some steps and it was a way to reward myself that happened in unplanned way. I would say reward in a sense that I was struggling a lot since I moved to [foreign country of study] struggling a lot and not having to every moment for a long time. just strolling through new language new culture no happy even though happy moment with the strangers but not with family. Got so much to get learn to learn more things about that environment I have to get to as from my family, because I wouldn't learn new culture if I was always in with my family. So it was also natural. It was not forced. But that happened once I came to [foreign country] also. I think after completion of [pre-university school], there was no satisfaction because I couldn't share everything with my family the way I would love to because everyone is busy. And after [pre-university school], there was no silly reason after getting University. No silly reason, but When I got to know that I'm moving to [2nd foreign country], and now I am done with two years, my two two years of my university already. I'm not quite sure if it was one year and a half. But yeah, of course, like it was in a vacation time. So yeah. I think it was a way to reward myself. Well, it wouldn't have happened if there was no funding from our uncle as well. But yeah, there was money, it was the minor aspect, because that's how the environment came there. But of course, like if I had to do my own, my own money would be an issue as well, like, everyone. Actually, they were like, several people like, around 10 people, during the same time from my family, some new ones who we weren't exactly my family, or their distant relatives, or some photographers, [home country] photographers. But, yeah, they went there, just to see, but all of them ended up doing it. So that was really fun. Yeah. I don't know, what was the question? Sorry? ... I think I said very nice about it. The meaning would be like, I think you should have rewards, so that you can think on new milestone. And I was thinking this, I was thinking it as a reward a way of rewarding myself, but also starting having a new beginning, I guess, I would say, because I was moving to new country from there. And I don't I don't have a concrete answer. But it just felt like I completed something. And I need to start something new. [E62] |
| Environment of Positive Emotion | And also, yeah, just trying not to be stressed out with everything because I know if I get stressed or like, you know, bothered, I think just do something like something fun, such as playing games and everything. So I think it helps me to be here. [E64] |
| Environment of Positive Emotion | It's, it's exciting and new for me. Because like, I've never experienced that, but then I have to assist them with that. So it is kind of exciting and also very new, like, I never deal with kids younger than primary school. So it is very meaningful, for me, at least. (E65) |
| Environment of Positive Emotion | That's why I don't know. Yeah, but it has. Yeah, but I would love it to be continued like to repeat this action, because I love this. Yes. (E70) |
| Environment of Positive Emotion | Meaning... I don't feel there's a deeper meaning it just fun to like, test each other. So I would say no deeper meaning (E82) |
| Environment of Positive Emotion | I don't think... it gives me a little bit of validation, like I'm doing something and it's productive. And, and it makes me. I mean, it's not that I live just for this but you know, when I wake up I have some something that I look forward to... What's going on? I Read news. (E87) |
| Environment of Positive Emotion | It was fun. Growing up. Yeah. (E90) |
| Environment of Positive Emotion | So this was a very fun, this is one of the most fun months, you know, one of the memories that you remember and cherish forever that this is how we actually met and interacted for the first time (E101) |
| Environment of Positive Emotion | it's very important for me, but these experiences are important for me. But I don't, like, I try not to think of these things to, like, a very deep extent. Like, I don't, like, try to bring out deeper meaning from something that doesn't have to have a deeper meaning, you know. So I wouldn't think so. That's, like, going, having fun, playing [sport], that was the only thing. (E109) |
| Environment of Positive Emotion | Because this experience, gave me the experience to this organization and, and made me fall in love with such actions, more and more. (E112) |
| Environment of Positive Emotion | And then the, the kind of recognition and satisfaction I get out of it as well. Both kind of important (E116) |
| Environment of Positive Emotion | Well, when you look back, I mean, a few weeks ago, just a couple of weeks ago, we celebrated our wedding anniversary and when you think about the history together you tend to remember these fond memories of playfulness. Of course, there are ceremonial moments, you know, the wedding there, these and that some traveling or whatever, but it is the good times that you mostly remember when looking back at the history together. Yeah. Okay, it represents the good times. (E124) |
| Environment of Positive Emotion | the meaning is that it's tradition. Yeah, it would be kind of, like established tradition, like religion. It's kind of cool. (E18) |
| Environment of Positive Emotion | Yeah. I think it was, it was pretty important in the way like, like I don't know. It's probably like. So that's why it's so meaningful. Like, yeah, like it's, it's like, a sign a name of, of, of my childhood games. So So Somehow that's, that's amazing (E114) |
| Environment of Positive Emotion | people usually think that a playful moment is usually the opposite of that. That it’s playful because it’s not important and they associate while I think people see it wrong. It’s playful because it’s not serious, but it can be playful and important. Serious I say in the sense of like grudging and like adult seriousness I mean. Because of course like for a kid football is very important or whatever thing their dolls are very important but in this sense that people associate that if its not like serious and mature then it’s not really important it’s just some fun or whatever but there was there was like there was a meaning on that for for us on that stage of our life and I think that because there was this meaning this connection with the space at the moment it was related to our university environment and graduation classes so it was like very representative of that phase of life and uh because of that um it kind of elicited all these moments because if it didn’t mean anything to us then it wouldn’t create an effect. (E1) |
| Personality Shift of Positive Emotion | It keeps bringing those feelings like back Yeah, because I could climb those you know, and actually like live in a hole in a whole nother like world that I created. I remember that I was like eight years old, and that was the best. Just being able to create those, like images. Even if there wasn't really anything around. It was like an old retirement like neighborhood but Never felt that way. It always felt like there was something else to find and explore" (E11) |
| Personality Shift of Positive Emotion | I think that actually, like really built our friendship up to that's it's still today, like we, we just cause shenanigans. We just do fun, silly stuff. Yeah. We're not like really close, personal friends who talk about deep things, but we always get together to do dumb stuff. (E17) |
| Personality Shift of Positive Emotion | I don't think it has like that much effect on my life. Playfulness in general feels like, makes me feel like First of all, it's like fulfilling in a way. It's fun. It's like stress relief, and it's sort of a will get away from your like, daily, just like normal adulting life. So yeah, living in general. does have like, positive, uplifting. You know, like the afterglow, I don't know how to explain it, but afterglow (E29) |
| Personality Shift of Positive Emotion | Yeah, it's just that kind of that situation and me realizing how much fun it is. influences the experiences that I seek out now in everyday life. So if I like any situation, I want to just have fun, close friends and have a good time with them. Like that's, that's what matters. So like, I just sit down and play a game with friends, like card game, or anything. That's always way more fun than just like, going out to some loud club or whatever. Just doing something else. Like I'd rather just find my people, my community, and just have fun with them." (E31) |
| Personality Shift of Positive Emotion | And it like really made me like back into that, like childish mindset of like, life is literally you can do whatever the fuck you want. Like, I think as you grow up, you think, Oh, I'm 18 I need to go to college and get a job, we need to get a car, but it's really you know, your life to live (E9) |
| Personality Shift of Positive Emotion | So in my life, I felt more confident and more outgoing person I think because I'm usually just playing games, if I know where I felt. It is okay to go outside and play with other people. (E50) |
| Personality Shift of Positive Emotion | I got to know, kids better, I got to understand. I mean, of course not really, like scientifically or academically or anything like the quality thing, but I just got to understand that they are so involved in anything you suggest, like whatever you offer they are in. At some point I understood, like maybe it's nice to live like that, to some extent, you know? Yeah. Like, I don't know what's going to happen, but I'm in. It sounds fun. Why not? You will never know until you get, like I'm feeling try and go through. So yeah, so maybe it's like, take it easy was taken exactly from that experience. And then, apart from that, I got a bit better, I got a bit a better understanding of this kind of like kid psychology, due to talks before or like, after, when I had some troubles during this play. (E52) |
| Personality Shift of Positive Emotion | Yeah, I mean, the meaning like, if you do something for the sake of just doing it, you will get like a deep joyfulness. (E69) |
| Personality Shift of Positive Emotion | Also, the meaning, when you use the word meaning, it seems strong. It seems like a strong word, a serious word. I don't like to be serious. Let me see. The meaning. I mean, enjoy. Enjoy. Enjoy the little things of life. Enjoy. Oh, I appreciate the little things of life. Participate in it. Be involved. Don't see people dancing. Don't see people being happy, and you're just there saying, oh, I was there. But be part of it. (E77) |
| Personality Shift of Positive Emotion | I'm not sure. I mean, I don't really think about it that much. Except, I mean, I thought of it because of this interview. I have remembered it over other incidents. And I think that's just because. Because I'm particularly proud of this one. Because I think, I think the execution of it went very well, compared to other little jokes here and there, or? Yeah. And it went on for a long time. And I involved another teacher. Yeah. I think just because, like, the scope of it was was bigger than most of the other similar kinds of things that were generally just like, over in 20-30 seconds. This went on. But in terms of meaning that it has on my life, I mean, not really much. I guess, like, I still think of it as an example of like, the kind of person I am. And, and the kind of teacher I am as well, like, just not always serious. I mean, you can, you can ask me who actually, like we used to work together at the first school that I worked at. And she wasn't a teacher but but she knew kind of what my reputation was, it's like a bit of a joke, but also like, like, I was really good at explaining grammar and stuff to like, other teachers would, would ask me for help if they didn't know how to explain a bit of stuff like that. So I used to really like teaching [name of person]. Quite difficult concepts and things like that, that students struggled with. So I don't know. Like, I think about things like that sometimes in terms of like, together with other memories. I have a teaching where Like, I know I'm a really good teacher, in terms of explaining grammar. I'm not very organized. That's, that's one area that I really fall down on. But I'm like, you can probably guess from my thoughts how they just went everywhere, but so like, on the one hand, I've got, like memories of like, I'm a good teacher, like, effective in terms of getting my students to understand things, but also fun. So like, I remember when I think about that day, I remember like, like, everyone was laughing. Like, yeah. So I guess I just had that in my head is like a little example of like, like, it's, it's definitely not, it's filed with like, the good memories of being a teacher like, not like, along with my failings, like all the times when I really like fucked something up. Like, it's, it's not in those, it's like, if I'm, if I'm having doubts about my abilities as a teacher, I've got like, the little folder of like, times I fucked up and the folder of like, things that I'm no good at. That's in the good folder, because I'm saying that, but I don't know. Anyway, I, I think I'm funny. Because my sense of humor matches my sense of humor. So, to me, I'm like, very funny. And whether other people find me funny is a different battle. Sometimes I'm the only one laughing at my jokes. But I remember in this case, everyone laughed. So some of my colleagues got a bit weird. But one of my colleagues, she found it really great. And so we had this talk together. So yeah, no, I don't know. It's a positive memory. And it helps me sometimes when I am, like doubting myself in terms of my abilities as a teacher. I guess. I feel like I'm, I'm only saying that though, because you're looking for an answer for what it means to be a burial life. And like, normally, I would say, not much. But I'm looking for meaning. And I guess that, that meaning is there, even if it's very weak? Like, I'm not basing my self worth as a teacher on this one joke, but it's there. I feel like God, buddy. Yeah. But it's not a very intense meaning. No, no, not at all. Not at all. I mean, I don't I don't often think of it. I thought of it because of because of this interview. Yeah, I have told I have told some friends about it. I think I think I generally remember things that I talked about as well. Like if I something happens that I never talked about it. Generally don't remember it very well. So it Yeah, I obviously have talked about this. So it has some some meaning, but I think probably only talked about in terms of like I did this funny thing in class once. Yeah. No. (E113) |
| Personality Shift of Positive Emotion | it gives me more pride I'm feeling proud that I have been through many and especially experiences and joyful experiences. So it's feeling proud Yeah, maybe maybe, when you have experienced many playful and joyful activities, you have become a difficult person to impress. If you have been if you have seen if you say that hypothetically, if you have seen everything in this earth, you will be impressed of all because everything is normal for you. This is for me one effect (E119) |
| Personality Shift of Positive Emotion | I think that it's that you can never take anything too seriously. Like, you can always find the positive. In any situation. Well, not really. But that sounds like a fortune cookie. I mean, it's like, you don't have to take Okay, let me say, let me put it this way. You don't have to take everything so seriously. Like, it might be a serious situation. But you know, you, you have to move on, you have to live on as hard as that can be. So for me, that kind of situation. tries to tell me like you can always, even though there is sometimes it's difficult to find, like a positive thing. You can always try to create a positive out of anything. (E123) |
| Personality Shift of Positive Emotion | This festival gave me and also appreciating nature. I think it was not only this festival, but this festival also puts the values in me for a reason to be nicer so yeah. [E63] |
| State of Lower Stress | when I think about like, why it was playful and why I enjoyed it was because it was like one of the first times One of the few times I had gotten out of my comfort zone, I was like, I'm going to do this with my friend because we're having fun and I want to keep having fun....I guess, like, pure child's like, or pure childness. Like, well, those moments where you think of kids, they're like, playing this what they do they play, not mindlessly, but they play in a way where nothing really matters. They are like, there's them their friends and fun. That's like they don't really there's like the You might consider consequences. But for the most part, they probably don't. They're just like, I'm going to do this because I want to have fun. And because my friends are doing it, and they want to have fun, so we're all going to do this. So that was like one of those moments, one of those pure, like, childlike moments that I've had of me like being happy having fun with my friend. And it's just also like, that unique experience that sort of makes it stand out. (E4) |
| State of Lower Stress | You can't do the serious stuff. Yeah. You just relax with a separation. (E7) |
| State of Lower Stress | I remember feeling such an innate sense of wholeness, and like fitting in, I guess. And just like camaraderie, and having fun and not caring, and all boundaries and walls, we're just kind of let down and everyone was on the same playing field. And we're just having fun. So we're laughing like crazy. .... I remember feeling very light. I remember not feeling guarded at all. I felt very. I wasn't comparing myself to anyone, which is something I think a lot of people in high school do. It's all about who looks like this, or how am I presenting, but it was everyone was just, you didn't care. Like you were? I don't know. Everyone was like the same person, if that makes sense. It was just I wasn't thinking about, Oh, am I messing up my lines? Because I was messing up their lines. I'm not thinking about how does my hair look? Am I like acting weird? Am I being too much? Because everyone was being too much or doing too weird. So it was just like a very physical sense of like, completeness, and no anxiety, no worry. Just kind of like energetic feeling, you know, like, very vibrant, (E8) |
| State of Lower Stress | Meaning it is finally done. Like now everyone knows that I'm [potentially socially risky sexual proclivity]. And I have a [partner] and which means I don't have anything to hide anymore. That's good. And when I put other photos on [social media], then I don't have to worry that people are seeing this [person] all the time. Who is that [person]? (E49) |
| State of Lower Stress | Well I think especially lately, It’s been really important because I’ve been going through some really grim times and I think having that made me feel really relaxed afterwards. it's I love playfulness. I love being in this difference reality state. I think for me in particular, because I've always had issues with depression and other stuff and then being able to get into the state where it's kind of like all that is gone for a while and then I’m just enjoying you know, just living in that moment. Without all of this weight of anxiety and depression and all those things. So I think playfulness is really meaningful to me (E56) |
| State of Lower Stress | It is also again, a moment when I felt I stopped focusing on... because I've been through so much anxiety as well. So it's the same thing. And it's like this moment of both. Yeah, but that and then I'm moving and I'm just doing something and just being there (E57) |
| State of Lower Stress | it was like my first year of [university]. And I knew the script. And he knew the script. And we were both just going off of each other.... And so finally, I just said, I was like, I don't want to watch a movie, you don't want to watch a movie. Let's just cut it out. Like, let's just have, like, let's just come here, what we came here to do, you know, we want to have fun, we want to have like a playful experience with each other, we want to have sex like we don't, you don't need to pretend (E12) |
| State of Lower Stress | And there's no rules how to behave. And yeah. And maybe three years. But also free to we will not worry about what happened after this, or the consequences about an illegal fireplace at the beach. (E13) |
| State of Lower Stress | I was controlled by other people all the time. And this was the period in my life when I started to make my own decisions and mistakes. And this was one of that experiences. Probably one of mistakes. I don't know. I don't think so. So it was like, for me, it was the beginning of my my own life. So I was taking the responsibility for what I'm doing. ....The meaning, I think is I've got a feeling that I can do something for myself. (E24) |
| State of Lower Stress | very light like an extra playful experience with game I would say. it allows me to do some kind of thing that doesn't feel like it was intended for or it's, it's a kind of something kind of surprising or novel. |
| State of Lower Stress | It's like a symbol of the teenager energy like you know Teachers and the parents think the teenager are supposed to be listen to their thoughts and be cute like be stay at their position and study study and Should leave whatever they want Parents want their kids live in their way So they're way they want so at that time we break the rule of ourself so because we are also come from that kind of family, so There's like a structure in our mind that we shouldn't do this. We should do this But at that point we feel that we are we are not living for them. We are living for ourself. We there's something we can do and We can cooperate with each other and we are not alone like before that we feel lonely because we just do what they want to do and we don't make much friends like we started well, and we if we started well, we teacher will oppress like oppress us or Conditional and I forgot the word did Like they will say some good words to us like and also give us some gifts But if we started very bad, then the teacher will call our parents to come to school Even if they know that parents has their work But the parents will come there because parents don't want students be kicked out from the school Yes, because it feel like a shame to be kicked out from school for the parents so and There's also another factor is the parents when the parents meet parents or their or for example the Talk whatever situation even if they just meet pass by or they eat together they will compare their child like my child has a very It's very listening to my words and very cute and study very well And and they feel proud of their child and if the children not study well, don't don't listen to their parents They will feel ashamed. They don't want to talk to their child so when I were other parents talk about their children and that parents will feel lonely and Then when they come back to face their child, they will punch their child like like really hate Really hate their child and and this is not illegal. This is totally going on in my country... We're in this context Where this kind of very high level of expectations, yes, we're here for you. This was something different than that. Yes This is something different Okay Let me broke the structure. They made for us and the structure just like a building a breakdown Then we see that the different perspective of the world not only the perspective of study hard and go to a high good university and Then find a stable job and live life like this and get married in the age of 26 and the 8th that that is what most of [Country] parents want their children to be because they think this is most safely because most of people do this and a day they leave and But but if there's but if someone don't do this, they will maybe live very badly so that is what their parents want the children do that the They think it's the most safe way to be alive Okay, and so it sounds like this this kind of set of expectations about what you should do and shouldn't yes It's almost like a full lifelong. Yes set of expectation. (E94) |
| State of Lower Stress | Hard to say it's doesn't have a really meaning. Beside have that kind of freedom ness. I have been, I'm saying that, but this side of it so it's, it's up to a kind of that's a you know, it's the freedom I'd say it's the freedom to say what one wants (E99) |
| Environment of Lower Stress | My body will be working but mind will sort of rest and refresh (E15) |
| Environment of Lower Stress | It's not a super important meaning. But it's Well, yes, it is important, because it's like the time we spend with my friends playing, I really enjoy. And it's like, it's the little moment of the week or time when I play that is like I there's not kind of obligations that small university or projects or work or problem is that like, two hours that we're playing and having fun. (E25) |
| Environment of Lower Stress | it helps me in kind of like to put balance, for example, on the days that I'm super stressed, as I said, I need to go out is like, okay, I obeyed for all the problems and I when I finish it like okay, I'm more like, also it has to, like it's one of the effects of running, I think, but as these experiences come when I run, I think it really did. Like it helped me a lot to evade my mind. And it's kind of like, Okay, I know, when you do a restart with a computer, sometimes it does say, I just started stopped to think so much. I just let my mind flow on the run, I finish activity and I start to think again, it's like, okay, now it seems better, .... when I go to run, running, it's kind of a boring activity. So my mind tends to kind of imaginer make it more playful to make it less boring (E26) |
| Environment of Lower Stress | It was quite profound. I want to slowly to get back into this kind of activity. But he also more than that, perhaps kind of reminds me of the baseline or that like maximum level? It gave me this idea of what is what is the maximum level and maximum enjoying experience without a cost? Because that's also part of it, maybe I could, I don't know, maybe, maybe I could do drugs and be more enjoy more. But But this has no price. So other than like, any other activity aside your life's economy, but it hasn't. It's not, there's no kind of hidden agendas, or there is no, nothing to be worried about it. (E37) |
| Environment of Lower Stress | it's more importantly, the sort of like, what I talked about the, you know, ridiculing this sort of like serious matters and whatnot. I think in this case, that's, that's a bit more important. Like you kind of like remind yourself that like, yeah, the political matters are important. But like, yeah, it's not life and death. (E39) |
| Environment of Lower Stress | And I think if they [video game characters] only make my free time, like meaningful, because after school and just kind of bored and don't know, what kind of thing to do, like the place where I live in the big city, and there's not much place for students to play. So I found that they made some kind of activity that made me feel relaxed and can stay alone without feeling lonely (E45) |
| Environment of Lower Stress | I think it gave me a very strong sense of safety and belonging. I found it to be a very kind of comforting presence. I found it to be a very I guess, you know, safe presence in my life, especially at that age and that stage in my life. (E47) |
| Environment of Lower Stress | but at the same time, probably, if, like, if I consider my, like, you know, choices before the concert, like this, abandoning this health situation, I would really say whether I need to do that or not, if I do it now, I mean, like, because it was kinda like, serious, I mean, the, the, the problem, and I need, I probably needed to, you know, like, quit the dance school for at least for some time. But that meant I will escape this last one, sorry. So, you know, value of, of probably, like, nowadays, value of that health condition and understanding what is really like important because now these stories is like done, is finished with the dancing stuffs. I mean, at least at that level, as it was at that time. So, this story is done, I'm still kind of like living good life, and finding any other, you know, you know, like activities to do. So, it's kinda like, diminishing these kind of, like, feeling of being a, what is it call, you know, like to sacrifice everything I had just to participate in this concert, I don't need anything, I will just ruin all I have, whatever it is, I will not read for my exams, I will see my costumes at night, I will do everything just to participate. It was kind of like, over the top to my mind now. So probably, in terms of sensibility, it's a bit of too much in terms of emotions that are derived and like, you know, yeah, it's amazing that it happened. And it's a bit of like, thoughtful, maybe, in terms of listening to yourself, because at that time, now, I'm kinda like, more sensible, and, you know, like, thinking that, yeah, probably, it shouldn't be, I should have done it in another way. But at that time, I was listening to myself. And probably it's also like, kind of, like, an example of the listening to myself in my situation. What works? Yeah, so this is, yeah, for the ---- very, very bright part, and very good to remember very, very amazing to have it in my life. But at the same time, very dubious. Rather, it like really what it was worth, worth doing that and, you know, abandoning everything, all these warnings and recommendations and, and stuff. (E53) |
| Environment of Lower Stress | And it was like all fun and games, I didn't have to worry about life. But yeah, it's comforting to know that, like, I could like, come home, and sit and just do that with people that I know and trust (E76) |
| Environment of Lower Stress | Makes me to force me to forget about the terrible experiences before and makes it makes me want to believe that there's someone better in the future. (E80) |
| Environment of Lower Stress | it's really important experience for me because I remember before we go into the amusement park, I was really stressful with all the schoolwork in my internship projects, but after that after doing The kinds of activities I feel like my stress are release. And I can face my reality more with more energy. (E81) |
| Environment of Lower Stress | Yes, it's I think you see influenced me because when I go down to the mountain I feel I feel like I'm full energy even if I'm I was really tired but I feel a lot of energy and I feel competent because all the bad thing is crushed by me and They're they're very easily crushed at that moment I can't cross them because I don't feel free. I don't feel there are somewhere I can go It's a kind of a limitation (E95) |
| Environment of Lower Stress | yeah, it's also one of those cases when you're stopped being in a place where you're obliged to be when when you start to be in places where you want to be. So that's also this kind of feeling of freedom, lets you do something. (E92) |
| Personality Shift Towards Lower Stress | gave me confidence. I found out that I can talk to people and we are all people. And we kind of are awkward. And it's, it's okay. It's happened. So it's like, not really confidence but being comfortable with being human.... It was a personal growth thing (E21) |
| Personality Shift Towards Lower Stress | I don't think it has like that much effect on my life. Playfulness in general feels like, makes me feel like First of all, it's like fulfilling in a way. It's fun. It's like stress relief, and it's sort of a will get away from your like, daily, just like normal adulting life. So yeah, living in general. does have like, positive, uplifting. You know, like the afterglow, I don't know how to explain it, but afterglow (E29) |
| Personality Shift Towards Lower Stress | In my life, maybe I feel more confident. Because actually, I'm a bit shy person. Really hard to get good the other friends was there I was so brave to talk to others people and talk to stranger and make friends and heard about others like kind of cultural nomads. So in my life, I felt more confident and more outgoing person I think because I'm usually just playing games, where I know I felt It is okay to go outside and play with other people. (E50) |
| Personality Shift Towards Lower Stress | The meaning, like, I think is really important. I think it kind of shaped my childhood. And like, um, yeah, if I did not have the chance to experience this, I think I would not be like this. Now. I start to, I think, start. Before I was playing the game, I was a little bit more. You know, me already, right. I'm not that scary. But when I was a kid, I was kind of, you know, scary. kind of, but then after playing this game, learning how to deal with losing the game and stuff. So I learned to regulate emotions. And I started to be more like fair and everything and like, if I'm angry, I would not be like exploding or something....And also, yeah, just trying not to be stressed out with everything because I know if I get stressed or like, you know, bothered, I think just do something like something fun, such as playing games and everything. So I think it helps me to be here. [E64] |
| Personality Shift Towards Lower Stress | I became a little bit more friendlier, a bit more social. And then I got a little less I don't know how to explain this, like little less scared of boys maybe. Basically, I became more socially adept. (E71) |
| Personality Shift Towards Lower Stress | Meaning, well, I sometimes believe we shouldn't take life too seriously. (E77) |
| Personality Shift Towards Lower Stress | I'm not sure. I mean, I don't really think about it that much. Except, I mean, I thought of it because of this interview. I have remembered it over other incidents. And I think that's just because. Because I'm particularly proud of this one. Because I think, I think the execution of it went very well, compared to other little jokes here and there, or? Yeah. And it went on for a long time. And I involved another teacher. Yeah. I think just because, like, the scope of it was was bigger than most of the other similar kinds of things that were generally just like, over in 20-30 seconds. This went on. But in terms of meaning that it has on my life, I mean, not really much. I guess, like, I still think of it as an example of like, the kind of person I am. And, and the kind of teacher I am as well, like, just not always serious. I mean, you can, you can ask me who actually, like we used to work together at the first school that I worked at. And she wasn't a teacher but but she knew kind of what my reputation was, it's like a bit of a joke, but also like, like, I was really good at explaining grammar and stuff to like, other teachers would, would ask me for help if they didn't know how to explain a bit of stuff like that. So I used to really like teaching [name of person]. Quite difficult concepts and things like that, that students struggled with. So I don't know. Like, I think about things like that sometimes in terms of like, together with other memories. I have a teaching where Like, I know I'm a really good teacher, in terms of explaining grammar. I'm not very organized. That's, that's one area that I really fall down on. But I'm like, you can probably guess from my thoughts how they just went everywhere, but so like, on the one hand, I've got, like memories of like, I'm a good teacher, like, effective in terms of getting my students to understand things, but also fun. So like, I remember when I think about that day, I remember like, like, everyone was laughing. Like, yeah. So I guess I just had that in my head is like a little example of like, like, it's, it's definitely not, it's filed with like, the good memories of being a teacher like, not like, along with my failings, like all the times when I really like fucked something up. Like, it's, it's not in those, it's like, if I'm, if I'm having doubts about my abilities as a teacher, I've got like, the little folder of like, times I fucked up and the folder of like, things that I'm no good at. That's in the good folder, because I'm saying that, but I don't know. Anyway, I, I think I'm funny. Because my sense of humor matches my sense of humor. So, to me, I'm like, very funny. And whether other people find me funny is a different battle. Sometimes I'm the only one laughing at my jokes. But I remember in this case, everyone laughed. So some of my colleagues got a bit weird. But one of my colleagues, she found it really great. And so we had this talk together. So yeah, no, I don't know. It's a positive memory. And it helps me sometimes when I am, like doubting myself in terms of my abilities as a teacher. I guess. I feel like I'm, I'm only saying that though, because you're looking for an answer for what it means to be a burial life. And like, normally, I would say, not much. But I'm looking for meaning. And I guess that, that meaning is there, even if it's very weak? Like, I'm not basing my self worth as a teacher on this one joke, but it's there. I feel like God, buddy. Yeah. But it's not a very intense meaning. No, no, not at all. Not at all. I mean, I don't I don't often think of it. I thought of it because of because of this interview. Yeah, I have told I have told some friends about it. I think I think I generally remember things that I talked about as well. Like if I something happens that I never talked about it. Generally don't remember it very well. So it Yeah, I obviously have talked about this. So it has some some meaning, but I think probably only talked about in terms of like I did this funny thing in class once. Yeah. No (E113) |
| Personality Shift Towards Lower Stress | I think that it's that you can never take anything too seriously. Like, you can always find the positive. In any situation. Well, not really. But that sounds like a fortune cookie. I mean, it's like, you don't have to take Okay, let me say, let me put it this way. You don't have to take everything so seriously. Like, it might be a serious situation. But you know, you, you have to move on, you have to live on as hard as that can be. So for me, that kind of situation. tries to tell me like you can always, even though there is sometimes it's difficult to find, like a positive thing. You can always try to create a positive out of anything. (E123) |
| Personality Shift Towards Lower Stress | The playfulness itself, I think, also worked as a tool to kind of build on my also my self confidence. When I felt like I was in a company that wouldn't judge me that would be welcoming and accepting of me and my ideas. I gradually started having more confidence in my ideas. And I started implementing my ideas outside of these spaces, for example in in like writing and things like that. (E47) |
| Personality Shift Towards Lower Stress | In my life, maybe I feel more confident. Because actually, I'm a bit shy person. Really hard to get good the other friends was there I was so brave to talk to others people and talk to stranger and make friends and heard about others like kind of cultural nomads. So in my life, I felt more confident and more outgoing person I think because I'm usually just playing games, where I know I felt It is okay to go outside and play with other people. (E50) |
| Personality Shift Towards Lower Stress | I got to know, kids better, I got to understand. I mean, of course not really, like scientifically or academically or anything like the quality thing, but I just got to understand that they are so involved in anything you suggest, like whatever you offer they are in. At some point I understood, like maybe it's nice to live like that, to some extent, you know? Yeah. Like, I don't know what's going to happen, but I'm in. It sounds fun. Why not? You will never know until you get, like I'm feeling try and go through. So yeah, so maybe it's like, take it easy was taken exactly from that experience. And then, apart from that, I got a bit better, I got a bit a better understanding of this kind of like kid psychology, due to talks before or like, after, when I had some troubles during this play. (E52) |
| Personality Shift Towards Lower Stress | The meaning, like, I think is really important. I think it kind of shaped my childhood. And like, um, yeah, if I did not have the chance to experience this, I think I would not be like this. Now. I start to, I think, start. Before I was playing the game, I was a little bit more. You know, me already, right. I'm not that scary. But when I was a kid, I was kind of, you know, scary. kind of, but then after playing this game, learning how to deal with losing the game and stuff. So I learned to regulate emotions. And I started to be more like fair and everything and like, if I'm angry, I would not be like exploding or something. So now I think this experiences have been really, really important to shape me at the moment. I think... And also shaped like me to be like, playful in general? Like, I like to socialize, maybe because I play a lot as well. And also, yeah, just trying not to be stressed out with everything because I know if I get stressed or like, you know, bothered, I think just do something like something fun, such as playing games and everything. So I think it helps me to be here. [E64] |
| State of Subjective Learning | And that's just like, I feel like it has this feeling of me Figuring out this new thing like that I think I should have known but I don't. And it's just like, looking at myself in the mirror and be like, wow. Wow, girl, we know. And you'll be like, Yeah, wow, I didn't know I would like that. But like, Hey, you like it? Yeah, I think I think like, it has that meaning. Like, I know what I like and what I don't like. And I know where I want to be in life. (E91) |
| State of Subjective Learning | Well, at least now I can look back and back to it and reflect on what I did. And then it seemed like, because it was ongoing, that I didn't really see the benefits of it. But now, it just, it was a great experience I learned. I learned a lot because the first time obviously, I didn't do well. And there were there were times when I didn't win as well, I lost and I felt like I let people down. (E97) |
| State of Subjective Learning | is a good example I've seen in a role playing game, and I tried to catch the same amount of attention and you know, having the choice or freedom for the player wise, in the games are, are around afterwards. And also when I was discussing about some video game mechanics with other people that played like, they could it's I will usually do like it could have felt like moments like this if offered some mechanics like this, or if they did this a bit differently. (E121) |
| State of Subjective Learning | I really appreciate that. Because, I mean, like, for many years before that I had not had the possibility to travel, because of the economy and everything. And I was expecting that for a long while. And then when it happened, and it came through, I feel like you you never come back home, the same person after you are going to a new place. And then I feel it changed the perspective somehow. Even though it's not like super far, it wasn't abroad, but, but it was something that most of [country of origin] and have the opportunity to do (E115) |
| Environment of Subjective Learning | You don't really have a time to stop and analyze. You just enjoy the role, right? You face the challenges. You have you're good and bad moments you get tired. You learn new things, but you never, never sit down and analyze. (E5) |
| Environment of Subjective Learning | Yes, it think it was one of most the best experience I had when. Yeah, definitely. No, because, you know, I'm not. I'm not living in the same country as [country of interview] which has nature everywhere. I'm living next to woods, of course, but I'm not so used to go into the woods, you know, you have woods, but it's not like oh, yeah, I'm going to the woods. Yeah. And so, in this kind of context nowadays, I'm going to do this kind of stuff regularly because of [hobby organization]. But before that I was not. Yeah, so it was something really unusual. Really. And I really liked that.... And doing games because I don't really like to walk for just walking. I prefer to do something nice with people. More than sports in itself, I don’t like sports, but I can easily just go and run and do sports if I’m with people and can have a game experience.... It’s not so much the woods, but nature. and I think this place is really amazing. It's, it's like you have the woods and you have also like, just like land with grass. Yeah. And there's the castle here. So it's, it's a really good place nice place was sunny and you know good weather. Yeah. So it's just like very good and close to home in the same time. So it's like something reachable. And in the center and unusual, I think it was really nice because usually something extraordinary is something totally different very hard. And this was in the same time very simple. But very exciting. (E6) [Nature] |
| Environment of Subjective Learning | It honestly made me realize that to talk to virtual characters, like their normal, like their regular people, I was like, I enjoyed that. I enjoy that. I enjoy pretending even though I know, it's just a game, I enjoyed pretending that it was more than it was. And I started kind of actively now looking for opportunities to sneak in some little, you know, bits of participation like that in there.... it changed a little bit, the way that I play the game. And I guess by extension, maybe other games (E27) |
| Environment of Subjective Learning | It was quite profound. I want to slowly to get back into this kind of activity. But he also more than that, perhaps kind of reminds me of the baseline or that like maximum level? It gave me this idea of what is what is the maximum level and maximum enjoying experience without a cost? Because that's also part of it, maybe I could, I don't know, maybe, maybe I could do drugs and be more enjoy more. But But this has no price. So other than like, any other activity aside your life's economy, but it hasn't. It's not, there's no kind of hidden agendas, or there is no, nothing to be worried about it. (E37) |
| Environment of Subjective Learning | Well, I think it's, to me, it's been a little bit about kind of expressing myself and like the my relationship with nature, like, being friends with the, you know, and in terms with the nature and kind of using what's in the nature to inspire myself, rather than just like, I don't know, destroying it. So kind of like, using what is in the nature to, you know, bring myself joy. So I feel I kind of like, made my relationship closer with that certain forest in a way, because like, I became more familiar with it. And I had these kind of, like, you know, fun, playful moments in it. So I got a flack, you know, got more attached to it in a way I feel. (E42) [Nature] |
| Environment of Subjective Learning | It's also not so much. I cannot say that it doesn't impact my life. we'd like it to sound like, like life changing. I'm not sure. I mean, since I played that game, I got more interested in like HP Lovecraft and his work in general, although, I mean, I bought the book. I bought the book with all his work, but I've not read it. So I don't know how useful it was divided. But yeah, I started to be more interested in in HP Lovecraft. Well, I don't know maybe it got me thinking about board games that are able to create kind of mysterious, maybe not horrifying, but like this kind of atmosphere. Do uncanny. Because usually, so I you know, I study horror video games. So I had not really thought about it so much related to board games. (E43) |
| Environment of Subjective Learning | it really happened a couple of days ago. But since a couple of days ago, I have been thinking about all the new ways that I can implement my new role as a [kink character]. And all the you know, I don't know. All the ways I can make this a thing. (E46) |
| Environment of Subjective Learning | I got to know, kids better, I got to understand. I mean, of course not really, like scientifically or academically or anything like the quality thing, but I just got to understand that they are so involved in anything you suggest, like whatever you offer they are in. At some point I understood, like maybe it's nice to live like that, to some extent, you know? Yeah. Like, I don't know what's going to happen, but I'm in. It sounds fun. Why not? You will never know until you get, like I'm feeling try and go through. So yeah, so maybe it's like, take it easy was taken exactly from that experience. And then, apart from that, I got a bit better, I got a bit a better understanding of this kind of like kid psychology, due to talks before or like, after, when I had some troubles during this play. I was calling some, like workers of this center for children's development and talk, like, whether it was my fault and how good I behave in a different way to make this real to ease in the situation. So I got a bit more knowledge. (E52) |
| Environment of Subjective Learning | Yeah, and I think being outside of normal, everyday situations allows you to see how people respond to stress and how they respond quickly, like there. But like the way I don't know what the word is, the way that people react. And I think that's a really true representation of somebody, when you see them react to something like, Okay, well, but they were thinking straight away when that happened. And of course, like, you can definitely stop and take it back and behave in a different way. But I think that's always really interesting to see that initial response from somebody. (E66) |
| Environment of Subjective Learning | And also, just me. I don't know, I think because of that I kind of grew up to be like, a little pranksters. Yeah, and yeah, I mean, like, a lot of things in my life. I connected with those experience, because I would, I played with people a lot growing up. Yeah, and just like, well, I played with people emotionally most of the time, because I'm just, like, making them confused. And just, and all that. And I think from that experience, just hearing those people just confused and like, like, lost and and all that. (E90) |
| Environment of Subjective Learning | whatever we experienced and how he trained us to actually talk to them [other gender people]. So this is this is a part of it, and this is why I remember it typically (E101) |
| Environment of Subjective Learning | Because this experience, gave me the experience to this organization and, and made me fall in love with such actions, more and more. Now, since my work is related, like managing the team, and managing resources, imagining my own startup and doing events over. So I think it had an effect of what I'm living today. (E112) |
| Environment of Subjective Learning | And then also, like, when you were doing it with other people. Because obviously, as you get older and older, you start doing it with your friends as well and kind of building, you'd sit there with all your Lego and build bits and pieces. So definitely. Yeah. Educational from the mechanical point of view, but also from a social point of view. (E117) |
| Environment of Subjective Learning | the idea of not drinking alcohol is also maybe to, like proving yourself and others, and that you can have fun and believe and be very playful and do all sorts of like really crazy stuff. Without the sort of use of alcohol. And then like, if, if you can prove that to yourself, and if you can prove that to others, then it might actually lead to a healthier lifestyle for you or others (E22) |
| Environment of Subjective Learning | That if people are all are older or something, it doesn't mean that they know how to do things better.... and also try and complete the new I have never done before. So I think this is important for me to to feel playful that I'm doing something I have never done before. Probably no one else around me have tried that already. It's a new thing. Cool. So both it's a new thing in your life, and it reinforced older people don't always know better. (E23) |
| Environment of Subjective Learning | I think that, that especially now starting [education of games], it's one of the things that I that I sort of go back to that, that made me that brought me where I am in a way because he my brother taught me all I know of video games up until I started playing on my own. So I feel like that's a big part of my sort of childhood. Like life as a gamer in general. So huge meaning (E34) |
| Environment of Subjective Learning | Well, the main meaning is that you should find, if you are a [country] or like a South Asian girl, you should find a husband who lets you be yourself, because we don't have any other way to get out of it, to be honest. I did try, just after my exams, I tried to go to Australia to do my bachelors and my parents were against it, like, you're a girl, alone, I can't let you go alone, I'll get married to somebody and go. So, this experience I should have had like a single person, I had to have that as a married person with a child, because my parents weren't allowing it. (E72) [Culutural symbol of how to play] |
| Environment of Subjective Learning | Um, I think at the, I think as I grew older, and I learned a lot like, I tried to, like, think back about this experience. It taught me a lot about dominance. Like, like, I press their numbers, so they have to answer. And I, I was in charge of the whole, like, experience. Like, they would have to answer whatever questions I have. So um, that taught me a lot of just like, controlling my whole, like, kind of like, place. (E90) |
| Environment of Subjective Learning | Meaning I personally believe that what I learned from this younger generation, and I'm also young, but more young, like teenage of the people who are in their teenage, I always like to interact with them to kind of be an elder that I didn't have growing up. So that they can talk to me openly and they can, you know, share things, if they're if something is troubling, then then they can share those things with me, I do this with my juniors as well, in college, in university or in when I was in college, so I like the, you know, the interaction that I have with the teenage group. So this, this actually is a very meaningful thing in my life, because I want to keep doing that, to inspire, you know, the younger people or to, you know, be there for them, to guide them. So yeah, this is this is a very strong meaning that I have for myself. And that I had and can get from this experience. (E103) |
| Environment of Subjective Learning | the meaning you gave me was that that you you you should do everything in proportions. That actually I think that gave me an info sample, okay, this time I needed to study and this time I needed to be with my family, for example, there was a time between the schools and also going out. So, there was a time then I would then I used to make sure that for example, if there is a grocery that I need to get from the market, so, anything that my family needs for the cooking and the you know, the important items. So, I used to make sure that I had after having a nap after after going to school and after having lunch, I used to have a nap. And after that, you know, I just wanted to make sure that okay, if there is any kind of work that my family needs to wants me to do while buying something or you know, fixing something. So, so, there was a progression and after that, for example, when there you know, you see there, the and after that I used to say okay, then after that, I used to feel that, okay, this is my time to play. And after, if I suppose this complete sunset, I used to come to my family. So I just read so after that, I said that was the part. So as I was with my friends, and after the sunset, we used to sit with the family and you know, having chit chats about everything, you know, making making jokes and you know, this stuff. And after that going to sleep and repeating the cycle. So I think that that particular spirit experiences gave me meaning about that you need to do you need to spend your life in proportion. It's like you also need to do your work, you also need your studies, you also need to be with your family, you also need to it is also equally important that you engage with your friends. So it's a kind of you for me that gives me meaning that your life is a wholesome experience of average doing everything in proportion... (E106) |
| Personality Shift of Subjective Learning | I guess the other thing that was really important to me, was that like, how to put it? I guess in some ways, it taught me also to be a sort of a little bit more spontaneous in this sense. (E38) |
| Personality Shift of Subjective Learning | gave me confidence. I found out that I can talk to people and we are all people. And we kind of are awkward. And it's, it's okay. It's happened. So it's like, not really confidence but being comfortable with being human.... It was a personal growth thing (E21) |
| Personality Shift of Subjective Learning | It honestly made me realize that to talk to virtual characters, like their normal, like their regular people, I was like, I enjoyed that. I enjoy that. I enjoy pretending even though I know, it's just a game, I enjoyed pretending that it was more than it was. And I started kind of actively now looking for opportunities to sneak in some little, you know, bits of participation like that in there.... it changed a little bit, the way that I play the game. And I guess by extension, maybe other games (E27) |
| Personality Shift of Subjective Learning | Yeah, it's just that kind of that situation and me realizing how much fun it is. influences the experiences that I seek out now in everyday life. So if I like any situation, I want to just have fun, close friends and have a good time with them. Like that's, that's what matters. So like, I just sit down and play a game with friends, like card game, or anything. That's always way more fun than just like, going out to some loud club or whatever. Just doing something else. Like I'd rather just find my people, my community, and just have fun with them." (E31) |
| Personality Shift of Subjective Learning | But also, I think it like, certainly gave me. I mean, it gave me this confidence, which is still carrying me to this day, academically, but like essays and stuff. But also like, I guess I saw it more of like, like appreciating art as well...just like it reigniting my passion for reading and analyzing stuff and whatnot (E75) |
| Personality Shift of Subjective Learning | This was the first experience for me to go outside my schedule. And it always has an impact, because I told you these trials. This is I remember this vividly because of myself alone. And after that. After that I we had similar experiences, again and again at different places. So and because now he's my fiance, I can't I can't stop it from having these kinds of experiences. So this is why now that experiences has a trickle down effect like a domino effect. Then we went for other things again, and again and again and again. And now have been doing it. Now I have to do it. So right experience as a base for me, where we started. We started as a couple and slowly transition into and slowly incorporating those kinds of experiences in my life. Now I have an experience that more often than I experienced it earlier. So this experience was a day for me to start with. (E100) |
| Personality Shift of Subjective Learning | See, I can definitely put a cube together. Well, I'm good. I'm one of the few people I know that doesn't seem to struggle with IKEA, which is beneficial? No, I think it's, it's definitely taught me a lot about being able to put things together, it's actually really helped in my jobs, I've ended up in the tech sphere. So it's, oh, most of it is like figuring out how this plugs into this is effectively like, how do we transport data? How do we manipulate it, etc. So it is that kind of lateral thinking through that educational piece of off, we do this, we do that. And especially, definitely the instruct, like the structured instruction based sets helped with that. But then it was, I think, a lot of that freeform on I'm going to make a rocket ship out of this plane, and this boat, and this, adding it all together, and then kind of making gear sets and stuff work. And Dad's an engineer as well. So he was always like, oh, yeah, if we do this, we do this, like we increase the ratio here to the gear ratio here, we'll put more force through this than that one, you turn this one a set number of times, etc, etc. So that basic kind of understanding of the natural world, and terms of mathematics and that sort of thing, as well as definitely been really, really beneficial. (E117) |
| Personality Shift of Subjective Learning | it was a moment, like, I felt like I could play a wide range of characters, because I was had like, a limiting factor of my place playing style and TTRPG, as was that I could not do play a few specific type of characters like, guys were straight, straightforward, and, you know, some kind of goody two shoes and, you know, to want to be hero, and so, and I could convince myself that I could also play other types of characters. So, it kind of gave me confidence in writing different types of characters to play. (E122) |
| Personality Shift of Subjective Learning | it changed my understanding and how I grasp reality. So well, when you have never been in a virtual space, you always think like, okay, like what we live right now is the only reality and that's it, there is nothing else. But once you once you once you have been in a virtual reality, you can also okay, like, what you feel what you are right now can change depending on how you change your change what you perceive. So, for example, we're sitting in this room and like in this like close space and stuff, but in the virtual reality, which could be someone completely different and it could still feel absolutely normal. It could be still a reality. Even though physically that is not it. But that's the whole point of the virtual reality. It's just like it's a reality that you create yourself. And you can do Even without a requiring any like VR headset and stuff, you can just like dream. It's like daydreaming, like you take yourself from the reality that you're physically in. And you go to this other reality where you do something you like.(E58) |
| Personality Shift of Subjective Learning | And after we became closer, I started to have some sort of realizations about my life. In a way, it helped me, it helped me have like a more, a better understanding of some things that I didn't know about me somehow. Like, I don't know, for example, let me think of something. I started to think about what I wanted to do, like after master's, I started to think about how I wanted like my apartment to be, stuff like that. But then again, it was not just that situation by itself. It was connected to how my relationship with this guy grew. Not because we became anything, but because we became so close through that experience that then later on, that support from his side helped me realize some other things. So in a way, it was like, we grew as, not as friends, but we grew as persons. Everyone, like he also had some realizations about stuff in his life. And so did I, in a very funny way. I don't know how or why it happened, but we, we grew. (E61) |
| Personality Shift of Subjective Learning | if I have to look into meaning, I don't know, meaning or the results, or maybe some kind of expectations, maybe some side effects that I learned a lot about myself. And I would say that I mean, I never thought the possibility of writing code and doing some some music, which was quite surprised for me. And yeah, I mean, if you're sometimes we're kind of looking into things and we don't understand much and later we discover that those things could be utilize mixed with something else you could do kind of new experience from that. So, I think the meaning could be meaning could be from this experience could be that I should look for the possibility in all ways always and in that I could make good have some skepticism. Being kind of skeptic for the even if things could be all the way right way. could be wrong. So you should be take care about that part. Also, that something bad could happen. Something good. could also happen. (E68) |
| Personality Shift of Subjective Learning | the importance of a work ethic and friendships mostly. So what goals Yeah, I'll just say those two the work ethic and the friendships. (E73) |
| Personality Shift of Subjective Learning | Okay, it help me realize that you can actually be a playful person and a serious person at the same time. But you just have to be able to balance them in a mature way, as a mature person. So it just depends on the way of your behavior, whether you could manage them both at the same time. (E79) |
| Personality Shift of Subjective Learning | It showed me that I’m a very hard working person and that I’m also committed. And that even though I might be the smartest guy on earth, but I compensate that with hard work and not giving up. It showed me a lot of things about myself. I knew them before but it proved them for me. (E86) [Hardworking] |
| Personality Shift of Subjective Learning | A lot. It has a lot of meaning. I don't know. I think like the most important part. I think the most important meaning is me. Figuring out me, like, like, yeah, who is this shallow person who is she and and I found a little like, I found a little bit about me there that I have zero clue about. (E91) |
| Personality Shift of Subjective Learning | That's my that's probably the just the continuation, which we've been talking about the feeling that if you're really wanting to do something, that if you would perform, put the effort to that that you can do that. (E92) [Hard work] |
| Personality Shift of Subjective Learning | yeah, I realized that I am good at this. You know this, because it was a job. So I realized that I'm good at creating it or giving sessions like this, and working for women rights. So that that kind of gave me the meaning and direction that where I want to be in my career. (E102) |
| Personality Shift of Subjective Learning | I can remember it that's a step towards believing that there is any sense any sense in guiding us... It feel free to say things and take it back or change your opinion there's no chisel, chisel and rock. The fact that it's this man was kind of a successful person in his own backyard, how he was building his own small Empire, how he his own son in the White House, etc, etc. I asked and at the same time, like talking with him for hours, he was telling me about his work as a translator in the Navy long time in targets, that he was also paid, because people his words were sales around. Things like that. Just getting getting a big term that I believe was the most of being somewhat accurate. Like biases. I would say that it did its part it was a one step towards this slow I would say distrust of authority or believing that authorities was the authority in house. that's accessible people. Probably just obsessive individuals. Okay, I'm not not.... No. It's, it's part in making me lose my belief in meritocracy. Okay, so it did its part in reducing my belief in meritocracy (E96) |
| Personality Shift of Subjective Learning | I think it gives me a kind of confidence to do at least try for things, which I think it is difficult. So I have learned a lesson that one should at least try what he is aiming for what he is thinking that he should do. So even though it seems very difficult in the start, or even it seems very daunting on the face, but at least one should try. (E104) [hardwork] |
| Personality Shift of Subjective Learning | This experience just reminds me that we can deal with the situation with every sort of situation we are facing, and never to go down. If you're right, in the wrong way, because we are sneaking out of those [rules] (E105) [freedom] |
| Personality Shift of Subjective Learning | I feel like you can improve yourself in that area. You can improve yourself in any other areas. It just takes discipline and put you put work into whatever you want to achieve or do or improve. So I think that (E107) [Hard work] |
| Personality Shift of Subjective Learning | It just taught me, like, my early experience that I described to you, it was very fun, and there weren't exactly a lot of rules. But when you're debating, you're supposed to follow a certain set of rules, that you can't exactly raise your voice, you can't talk out of turn, and stuff like that. So I would say it brought a certain discipline, I would say, in my life. I don't think I would intentionally interrupt someone when they're speaking. I think, yeah, so it brought a certain amount of discipline in my life, in my day-to-day life. (E108) [Hardwork] |
| Unclear State | was not particularly significant, because I haven't had a think about that particular event until you've asked about it. So like, it's probably not all that significant in and of itself, like, probably just like, fine at the time. Yeah. (E118) |
| Unclear Environment | Just a past time as a kid (E74) |
| Country | Number of Respondents |
| Brazil | 2 |
| Spain | 3 |
| Finland | 10 |
| USA | 6 |
| Turkey | 3 |
| Bulgaria | 1 |
| Belarus | 1 |
| France | 3 |
| Mayotte | 1 |
| Germany | 1 |
| Russia | 5 |
| Mexico | 3 |
| Nepal | 3 |
| Portugal | 1 |
| Czech Republic | 2 |
| Romania | 1 |
| Switzerland | 1 |
| Canada | 1 |
| Thailand | 1 |
| Hong Kong | 1 |
| South Korea | 2 |
| Iran | 1 |
| Ethiopia | 1 |
| Indonesia | 2 |
| Australia | 4 |
| New Zealand | 1 |
| Pakistan | 7 |
| Palestine | 2 |
| Taiwan | 1 |
| China | 1 |
| Azerbaijan | 2 |
| Sri Lanka | 2 |
| Egypt | 2 |
| Kenya | 1 |
| Colombia | 1 |
| Nigeria | 2 |
| Andora | 1 |
| Vietnam | 1 |
| Cameroon | 1 |
| El Salvador | 1 |
| India | 1 |
| Lebanon | 1 |

**Interview Protocol**

**Demographics**

Please answer the following questions about yourself.

Age

Gender

Country of Nationality

Native language

Highest education level

Current Occupation

Culture

When you were growing up, where were you living?

Where else have you lived throughout your life?

How would you briefly describe the cultures you have lived in?

Would you say your upbringing was culturally similar to people from the same nation? How so? Any differences? (How would you describe the cultural norms of the location you grew up in?)

Are there any other identifiable groups that you would consider yourself culturally connected to?

Are there any other cultures you’ve lived or know intimately that feel different than the culture you grew up in? How would you describe these cultures?

**Introduction**

This interview is asking you questions about playfulness. For the use of this interview please think of playfulness as the internal experience often associated with play.

Now, imagine playful experiences and try to think of one of your most playful experiences. Because this is an internal experience you could be doing any type of activity as long as you were feeling or thinking in a highly playful way. There is no right or wrong answer. Don’t rush your answer, if nothing comes to mind immediately, relax and take a moment and reflect.

Once you have a specific example in mind, please recall the sensations, feelings, and thoughts you experienced. Respond to the questions based upon your personal experience.

Please describe the playful experience in detail, focusing on the way in which you perceived and interpreted the event:

*Section 1 Occupational Context :*

Who were you with during this experience?

Were they an important part of your experience? In what way?

Where were you during this experience?

At what time did this experience occur? If you were to define when this experience

“started” and “stopped” when did this experience start and stop?

How long ago was this experience?

What activity were you primarily engaged in during this experience?

Is this an activity you had done before?

How many years have you done the activity?

How frequently per week would you do this activity?

What other activities were you engaged in during this experience?

*Section 2 Cognition:*

What kinds of physical or bodily sensations were occurring during this experience?

What kinds of emotions were you feelings?

What kinds of thoughts were you having?

What were you paying attention to the most in this experience?

What kinds of larger aspects of your life do you think were connected to the playfulness of this experience?

*Section 4 Motivation/Goal:*

Why were you engaged in this activity originally?

When you were in the experience, what was motivating your choices?

Why did this experience stop?

Over all, why do you think this was such a playful experience?

If you were to list all of the important factors that enabled this experience to be as playful as it was what factors would you list?

1.

2.

3.

4.

5.

(feel free to add more factors or leave some numbers blank)

If you were describe this experience using single words other than the word playful, how else would you describe this experience?

What is the meaning this experience has had in your life?

If you could, would you repeat this experience?

Looking back at the experience do you support the decisions you were making?

*Section 6 Game Studies Questions*

Did you feel like there were rules during this experience?

Were any of the rules non-playful?

Were there any rules that were normally present that were not present during this experience?

Were changing or creating any rules playfully during this experience?

Were there any rules that were playful to follow?

Did you bending or breaking any rules playfully during this experience?

Did you feel capable or skilled during this experience?

Were you seeking greater skill or competence during this experience?

Do you feel like you were the source of what was happening during this experience?

Do you feel like something outside of you was the source of what was happening during this experience?

Did you feel related to the people around you during this experience?

Did you feel like you had the freedom to act upon what you wanted in this experience?

Did you feel like normal reality was suspended during this experience?

How much were you considering your choices during this experience?

Do you feel like you chose to have this experience?

Did this experience feel challenging to you? In what way?

Do you feel like you had the appropriate skills to face these challenges?

Was the Process of this experience more important than the end result or the end result more important than the process?

Please respond to the following words or short phrases in terms of how well you feel they describe your experience. Please answer with a number on a scale of 1-7 where

1= “Strongly Disagree”

2= “Disagree”

3= “Slightly Disagree”

4= “Neither Agree nor Disagree”

5= “Slightly Agree”

6= “Agree”

7= “Strongly Agree”

**Phrase** **Answer** (Scale 1-7)

This experience was *Playful*. ____

This experience was *engaging*. ____

I felt *open* during this experience ____

I was making *offers* to make this experience more engaging for myself ____

Or other around me during this experience

I was engaging with *non-real or imaginary* things during this experience. ____

I was engaging in an *unconventional manner* during this experience ____

I was engaging *for the sake of how it made me feel, without considering ____*

*other consequences*

During this experience...

I was *socially active*  ____

My *emotions* were making me want to continue or go deeper into the experience ____

I was *actively thinking* ____

I was *physically active* ____

This experience included

Discovery-

Creativity-

Games- ____

Humor- ____

I would describe this memory as including the feeling of...

Vivid Fantasy ____

Celebration/partying ____

Play with objects / location ____

Flirtation ____

Seeking a desired outcome ____

Transgression ____

I would describe the following as important for the experience that occurred

Location of the experience _____

People involved in the experience _____

Purpose or goal in the experience _____

Activity during the experience _____

The culture I was in during the experience _____

How do you think your culture affected this experience? (No longer numbers)

What would be your culture’s general view of this experience?

*Section 7 General Questions*

Are there any important parts of this experience or how you interpreted it that you feel

like you have not discussed?

What other experiences were you thinking about discussing for this interview?

Would you like to be interviewed again focusing on one of those experiences?

*Section 8 Playfulness*

When you were thinking about playfulness, did you try and translate playfulness into any other language? What word did you use? What word would you use to translate playfulness into your native language and any other language you are fluent in?

What is the context you would normally use this word?

Are there differences between how you would use that word and playfulness?

How would you generally define the concept of playfulness?

If you were to name 5 words in english that you associate with playfulness what words would you choose?

How would you say your culture affects your viewpoint of playfulness?

What is the general perspective on playfulness from your culture?

In your culture, are there any boundaries of when you shouldn’t be playful?

In your culture, are there any types of playfulness you would consider expected or common?

What are the general consequences for playfulness in your life?

Have you had any changes in how playful you are throughout your life?

Short Measure of Adult Playfulness

Would you please answer how well you think the following statements describe you in your normal day to day life.

(4 point scale) (1=Strongly disagree, 2=slightly disagree, 3= Slightly agree, 4=strongly agree)

1 = *“I am a playful person*”;

2 = “*Good friends would describe me as a playful person*”;

3 = “*I frequently do playful things in my daily life*”;

4 = “*It does not take much for me to change from a serious to a playful frame of mind*”;

5 = “*Sometimes, I completely forget about the time and am absorbed in a playful activity*”.
